# An Abscopal Effect on Lung Metastases in Canine Mammary Cancer Patients Induced by Neoadjuvant Intratumoral Immunotherapy with Cowpea Mosaic Virus Nanoparticles and Anti-Canine PD-1

**DOI:** 10.3390/cells13171478

**Published:** 2024-09-03

**Authors:** Petra Sergent, Juan Carlos Pinto-Cárdenas, Adhara Jaciel Arreguin Carrillo, Daniel Luna Dávalos, Marisa Daniela González Pérez, Dora Alicia Mendoza Lechuga, Daniel Alonso-Miguel, Evelien Schaafsma, Abigail Jiménez Cuarenta, Diana Cárdenas Muñoz, Yuliana Zarabanda, Scott M. Palisoul, Petra J. Lewis, Fred W. Kolling, Jessica Fernanda Affonso de Oliveira, Nicole F. Steinmetz, Jay L. Rothstein, Louise Lines, Randolph J. Noelle, Steven Fiering, Hugo Arias-Pulido

**Affiliations:** 1 Department of Microbiology and Immunology, Geisel School of Medicine at Dartmouth, Lebanon, NH 03756, USAjanet.lines@dartmouth.edu (L.L.); randolph.j.noelle@dartmouth.edu (R.J.N.); steven.n.fiering@dartmouth.edu (S.F.); 2 DIAGSA, Diagnostico de Salud Animal, Naucalpan 53910, Mexico, Mexico; juancarlospintoc@gmail.com; 3 Centro Veterinario Valles, Zapopan 45070, Jalisco, Mexico; adhara.jaciel.arreca@gmail.com (A.J.A.C.); azul_vet8@hotmail.com (M.D.G.P.); alicia_mendoza_14@hotmail.com (D.A.M.L.); aby_270691@hotmail.com (A.J.C.); card_dia@hotmail.com (D.C.M.); 4 VETCONNECT Diagnóstico por imagen, Via Toledo, 2952 Mas Palomas, Monterrey 64780, Nuevo León, Mexico; drvet.daniel.luna@gmail.com; 5 Department of Animal Medicine and Surgery, Veterinary Medicine School, Complutense University of Madrid, 28040 Madrid, Spain; danialon@ucm.es; 6 Aquila Data Analytics, LLC., Concord, NH 03766, USA; info@aquiladataanalytics.com; 7 Lab for Vets, Zapopan 45086, Jalisco, Mexico; yzarabanda@hotmail.com; 8 Department of Pathology and Laboratory Medicine at Dartmouth Hitchcock Health, Center for Clinical Genomics and Advanced Technology, Lebanon, NH 03756, USA; scott.m.palisoul@hitchcock.org; 9 Department of Radiology Dartmouth Health Geisel School of Medicine, Lebanon, NH 03755, USA; petra.j.lewis@hitchcock.org; 10 Dartmouth Cancer Center, Geisel School of Medicine at Dartmouth, Lebanon, NH 03756, USA; fred.w.kolling.iv@dartmouth.edu; 11 Aiiso Yufeng Li Family Department of Chemical and Nano Engineering, University of California San Diego, 9500 Gilman Dr., La Jolla, CA 92093, USA; jaffonsodeoliveira@ucsd.edu (J.F.A.d.O.); nsteinmetz@ucsd.edu (N.F.S.); 12 Moores Cancer Center, University of California San Diego, 9500 Gilman Dr., La Jolla, CA 92093, USA; 13 Center for Nano-ImmunoEngineering, University of California San Diego, 9500 Gilman Dr., La Jolla, CA 92093, USA; 14 Shu and K.C. Chien and Peter Farrell Collaboratory, University of California San Diego, La Jolla, CA 92093, USA; 15 Department of Radiology, University of California San Diego, 9500 Gilman Dr., La Jolla, CA 92093, USA; 16 Department of Bioengineering, University of California San Diego, 9500 Gilman Dr., La Jolla, CA 92093, USA; 17 Institute for Materials Discovery and Design, University of California San Diego, 9500 Gilman Dr., La Jolla, CA 92093, USA; 18 Center for Engineering in Cancer, University of California San Diego, La Jolla, CA 92093, USA; 19 Lifordi Immunotherapeutics, Lebanon, NH 03756, USA; jay.l.rothstein@dartmouth.edu

**Keywords:** canine mammary carcinomas, intratumoral injections, plant virus, cowpea mosaic virus, immune cells, tumor microenvironment, anti-canine PD-1, abscopal effect, lung metastasis, canine NanoString array

## Abstract

Neoadjuvant intratumoral (IT) therapy could amplify the weak responses to checkpoint blockade therapy observed in breast cancer (BC). In this study, we administered neoadjuvant IT anti-canine PD-1 therapy (IT acPD-1) alone or combined with IT cowpea mosaic virus therapy (IT CPMV/acPD-1) to companion dogs diagnosed with canine mammary cancer (CMC), a spontaneous tumor resembling human BC. CMC patients treated weekly with acPD-1 (n = 3) or CPMV/acPD-1 (n = 3) for four weeks or with CPMV/acPD-1 (n = 3 patients not candidates for surgery) for up to 11 weeks did not experience immune-related adverse events. We found that acPD-1 and CPMV/acPD-1 injections resulted in tumor control and a reduction in injected tumors in all patients and in noninjected tumors located in the ipsilateral and contralateral mammary chains of treated dogs. In two metastatic CMC patients, CPMV/acPD-1 treatments resulted in the control and reduction of established lung metastases. CPMV/acPD-1 treatments were associated with altered gene expression related to TLR1–4 signaling and complement pathways. These novel therapies could be effective for CMC patients. Owing to the extensive similarities between CMC and human BC, IT CPMV combined with approved anti-PD-1 therapies could be a novel and effective immunotherapy to treat local BC and suppress metastatic BC.

## 1. Introduction

Breast cancer (BC) remains the most common cancer in US women, with ~298,000 estimated new cases in 2023. BC is the second leading cause of cancer deaths in the US, with ~43,200 estimated deaths in 2023, despite multidisciplinary treatments, including chemotherapy, surgery, radiation, and, when appropiate, targeted therapy [1]. Anti-PD-1 immunotherapy for solid tumors has a wide range of efficacy. While practice-changing results have been observed in mismatch repair-deficient, locally advanced rectal cancer [2], its efficacy is modest for most tumors, including BC [3,4,5,6]. There is an urgent need for strategies that can alter the immune-suppressive tumor microenvironment (TME) and increase the efficacy of anti-PD-1 therapy in BC patients.

The development of immunotherapies for BC is hindered by the lack of optimal preclinical models with biological and immunological features similar to those of humans, which, if available, would enable better evaluation of novel treatments. We and others have demonstrated that companion dogs with spontaneous canine mammary cancer (CMC) share clinicopathologic, genomic, and immunologic features with human BC patients [7,8,9,10,11,12,13,14,15,16,17,18]. These characteristics in animals with large tumors make them a uniquely valuable heterogeneous animal population to evaluate the clinical efficacy of new immunotherapeutic agents and their combinations with high confidence that findings will predict human clinical trial outcomes.

We previously demonstrated that intratumoral (IT) therapy with cowpea mosaic virus nanoparticles (CPMV), a plant virus that does not infect animals, led to strong immunostimulatory signaling through toll-like receptors (TLRs) 2, 4, and 7. When delivered intratumorally, CPMV converts cold tumors into hot tumors by activating innate immune cells which release pro-inflammatory cytokines that stimulate local and systemic T cell anti-tumor immunity [19,20]. Our previous studies in ovarian, colorectal, and melanoma mouse models demonstrated that IT CPMV significantly increased PD-1 levels in Foxp3^−^CD4^+^ effector T cells and CD44^+^CD8^+^ effector T cells [21], implying that the strong immunostimulatory properties of CPMV could synergize with anti-PD-1 therapy. Indeed, combining IT CPMV with systemic anti-PD-1 increased the total number of CD4^+^ and CD8^+^ T cells; their effector memory subsets (CD44^+^CD62L^−^); the CD8^+^/regulatory T cell ratio; and the proportion of NK cells [21]. Overall, treatment elicited a long-term immune response by generating systemic tumor-specific T cells and increased survival in these murine models [21].

It should be noted that human patients receive systemic (intravenous; IV) anti-PD-1 therapy. However, in this study, we propose IT anti-PD-1 therapy for the following reasons: First, systemic anti-PD-1 therapy is linked to immune-related adverse events (irAEs) in both human and canine cancer patients, including therapy-related death [22,23,24]. In contrast, IT treatment has not been associated with serious irAEs in human cancer patients [25,26] or in canine patients treated with anti-PD-1 (our preliminary data), other IT treatments like IL2/IL12 in canine soft tissue sarcoma and melanoma patients [27,28], or anti-OX40 combined with a toll-like receptor (TLR3/8) agonist in various canine solid tumors [29]. Second, IV anti-PD-1 administration requires a large amount of anti-PD-1, which can lead to both biological toxicities, including death [22], and financial challenges associated with the high cost of the large doses needed to systemically treat dogs. The IT route requires lower amounts of drugs, significantly decreasing the high cost linked to IV immunotherapies [30], and is associated with a reduced risk of irAEs by lowering systemic exposure while maintaining efficacy on injected lesions. Third, IT therapy, including anti-PD1, has been applied to human BC, demonstrating feasibility, safety, good clinical responses, and low irAEs [25,26,31]. Lastly, it should be highlighted that the IT procedure for accessible superficial tumor masses is a simple and straightforward, minimally invasive procedure requiring minimal equipment, and can be performed in the outpatient setting by a physician or veterinarian with minimum training [26,32]. In our hands, it takes 3–5 min to carefully administer IT drugs to CMC patients. 

We have demonstrated that IT CPMV treatments are safe and effective in CMC patients independent of clinical stage, tumor size, histopathologic grade, and tumor subtypes [33,34]. To model current PD-1-based immunotherapy in BC [4,35,36] and accelerate the implementation of human clinical trials with IT CPMV, we developed a monoclonal mouse antibody against canine PD-1 (acPD-1) and applied IT acPD-1 as a monotherapy or as a combined therapy with IT CPMV (IT CPMV/acPD-1) in six female CMC patients. Our study demonstrates that acPD-1 monotherapy and combined CPMV/acPD-1 therapy are safe, well-tolerated, and do not cause irAEs; have a positive effect on controlling tumor burden in injected and noninjected tumors; and have an impact on established lung metastases using CPMV/acPD-1.

## 2. Materials and Methods

### 2.1. Canine Patient Recruitment and Selection Criteria

CMC is rare in the US because female dogs are generally spayed when young. This is not the case in many other countries which do not aggressively spay young female dogs. This prospective proof-of-concept, open-label study was performed at Centro Veterinario Valles, Zapopan, Jalisco, Mexico, and DIAGSA, Naucalpan, Mexico. All patients’ owners signed an informed consent form. This study is approved by the Internal Committee for the Care and Use of Animals, Faculty of Veterinary Medicine and Zootechnics of the National Autonomous University of Mexico (Protocol #153). Client-owned dogs with histologically confirmed diagnosis of mammary gland cancer with a tumor mass of at least 1.5 cm in any length, with or without metastatic disease, were eligible for enrollment. Prior chemotherapy, radiation therapy, or other investigational drugs were not allowed (additional details are described in the Supplemental File). The characteristics of six individual dogs are described in Table 1 and Table S1. The clinical staging system, histopathological classification of tumors, and the histological grade of malignancy were evaluated as described elsewhere [37,38,39].

### 2.2. Safety Evaluation

Hemograms and biochemistry analyses were performed weekly to evaluate systemic changes and track irAEs (additional details are described in the Supplemental File). After IT injections, each canine patient was closely observed by the attending veterinarian for approximately four hours in the veterinary clinic with a follow-up three days later in the clinic. In addition, the dogs were observed daily by their owners to detect possible irAEs using a preestablished quality of life (QOL) survey [40], which was reviewed by the attending veterinarian prior to planned weekly treatment. The evaluation of hematological, biochemical, and other adverse events related to immunotherapy was conducted according to the Veterinary Cooperative Oncology Group criteria (Version 2) [41]. 

### 2.3. Study Design

The primary objective of this open-label study in CMC patients was to determine the safety profile, tolerability, and dosage of neoadjuvant IT acPD-1 therapy as a monotherapy or in combination with IT CPMV (CPMV/acPD-1) therapy in CMC patients. Secondary endpoints evaluated the overall response rate (ORR), immune-related adverse events (irAEs), and quality of life (QOL). 

**Figure 1:**
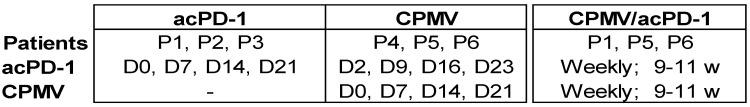
Layout of the trial with neoadjuvant intratumoral ac-PD1 and CPMV/ac-PD1 injections. Before treatment, patient evaluation included the collection of a blood sample, thoracic radiographs, tumor measurements, QOL evaluation, and collection of an FNA. During a four-week treatment period, three dogs (P1–P3) received IT acPD-1 weekly and three dogs (P4–P6) received IT CPMV (D0) and, two days later, IT acPD-1 (D2). Three dogs (P1, P5, and P6) were treated further after D29 with CPMV/acPD-1 as a single weekly IT injection for 9 to 11 weeks.

The largest tumor mass in each patient was selected as the target tumor (injected) for IT injections. Other mammary nodules present in the same and contralateral chains were observed to evaluate the systemic impact on noninjected nodules in the same canine patient. Similarly, thoracic radiographs were used to track the abscopal effect on lung metastases in patients P5 and P6, who had metastatic lung disease at diagnosis. The scheme of the trial is illustrated in Figure 1. CMC patients were randomly enrolled in the acPD-1 arm (P1–P3) and the CPMV/acPD-1 arm (P4–P6). The acPD-1 treatment arm consisted of IT acPD-1 weekly. The combination therapy CPMV/acPD-1 arm consisted of weekly IT CPMV followed by IT acPD-1 two days later. 

This weekly treatment was administered for four weeks. After the four weeks of treatment, patients P2, P3, and P4 underwent planned surgery with adjuvant therapy as recommended (described in the Supplemental File). Surgical procedures were performed according to the institutional standard of care protocol (described in the Supplemental File). Patients P1, P5, and P6 continued on the combination therapy at D29 because they were not candidates for surgery (Figure 1): P1 suffered from spondylosis deformans and other comorbidities, limiting surgery for her; P5 and P6, two metastatic patients, were in good health at D29 and the combined immunotherapy was having a positive effect on their tumors and lung metastases. Hence, they continued with weekly IT CPMV/anti-PD-1 treatments. Based on hemogram and biochemistry data and the tumor response at D29, the IT doses for the combination therapy were 0.4 mg of CPMV and 0.5 mg of acPD-1 from this day onwards. 

### 2.4. Treatment

CPMV nanoparticles were produced in plants as described previously [42], and the IT CPMV dose of 0.2 mg was used as in previous CMC studies [33,34]. Depending on the tumor volume, the CPMV dose was administered in 0.3 to 0.8 mL of sterile phosphate-buffered saline (PBS) and injected at two to four different sites of the selected target tumor using a 25G needle. The acPD-1 antibody is a mouse anti-canine PD-1 produced by immunizing mice with purified recombinant canine PD-1 to create hybridomas, which were then screened to generate five candidate monoclonal antibodies. The lead monoclonal antibody was used here, and the full characterization of the antibody will be described separately (Noelle and Arias-Pulido, manuscript in preparation). For the acPD-1 dosage, we used a modified version of the accelerated titration design [43] wherein single-patient cohorts with double-dose escalation steps were treated. Based on published clinical trials in canine cancer patients using systemic anti-PD-1 (3 mg/kg) [22], we allometrically scaled the dosing to dogs weighing 5 kg to 30 kg and used 1/100th of the systemic dose, which gave a range of 0.15 to 0.90 mg per single injection. The starting IT acPD-1 dose was 0.125 mg and increased weekly to 0.25 mg, 0.50 mg, and 1.00 mg. For the IT CPMV/ac-PD-1 dose, the same acPD-1 dose escalation was applied with a fixed CPMV dose of 0.2 mg. The acPD-1 dose in PBS was applied as described above for the CPMV dose. For the combined treatment, the CPMV dose was mixed with the acPD-1 dose and administered as a single IT injection, as described above. P5 had two target tumors (P5.1 and P5.2), each treated with an individual IT dose. No attempts were made to avoid necrotic tumor regions.

### 2.5. Tumor Response Evaluation

The tumor response to the IT treatment was evaluated once or twice a week during the treatment period by measuring the tumor volume (Tv) using the formula Tv = 0.5*long axis*(short axis)^2^. The injected tumor and any uninjected mammary nodules in the same or contralateral chains were evaluated in a similar manner. The percentage of tumor growth inhibition (%TGI) was estimated as %TGI = 100 × (final Tv − initial Tv)/initial Tv. All measurements are in cubic centimeters (cm^3^). Taking D0 as the reference, responses were defined as complete response (CR) when there was a disappearance of all target lesions; partial response (PR), when at least a 30% decrease in the target lesions occurred; progressive disease (PD), when at least a 20% increase in the target lesions occurred or one or more new lesions appeared; and stable disease (SD), when neither sufficient shrinkage to qualify for PR nor sufficient increase to qualify for PD occurred. ORR was defined as CR+PR. In addition, for exploratory analyses, we applied the itRECIST criteria [44]. The clinical benefit (CB) in humans is defined as CR+PR+SD for at least 6 months from the best response date. For the CB in dogs, and considering the dog’s age, size, and weight [45], the 6-month life period in humans translates to roughly one month in a dog’s life. 

In dogs, three-view thoracic radiography, consisting of the right and left lateral and ventrodorsal or dorsoventral views, is standard for detecting pulmonary metastases [46] and each patient had three-view radiographs. Radiographs were digital with a direct system (Xmaru model DR, Rayence, Gyeonggi-do, Korea). A grid (>10 cm in thickness) was used in all patients. All radiographs were assessed as being of adequate diagnostic quality. The interpreters (Dr. DLD, a veterinary expert on canine radiographs, and Dr. PJL, an expert on human breast radiographs) were blinded to the patient’s information. All three radiographs were interpreted separately with the final call made by Dr. DLD. The location (lung lobe affected) and size of the pulmonary nodules were recorded using images in Digital Imaging and Communications in Medicine format and analyzed with the HOROS software (https://horosproject.org/; accessed on 30 June 2024). 

### 2.6. Histopathology and Immunohistochemistry (IHC) Assays

Single 4 mm tumor tissue sections were used for histopathology and IHC assays for estrogen receptor, progesterone receptor, and human epidermal factor receptor-2 (HER2). The IHC details are described in the Supplemental File and Table S2. 

### 2.7. Transcriptome Analysis of the TME

An FNA of the target injected tumor was taken from the three dogs in the acPD-1 arm and the three dogs in the CPMV/acPD-1 arm before the intratumoral injections. On the surgery day, additional FNAs and tumor samples from surgical specimens were collected. Lung metastatic samples were collected at necropsies. FNA samples were stored in RNAlater solution (Qiagen, Germantown, MD, USA) at −80 °C until they were processed. Surgical biopsies and lung metastatic samples were collected and stored in buffered formalin, and embedded in paraffin. RNA was isolated from FNA samples using the RNAeasy Plus Micro Kit and the RNEasy FFPE kit was used for paraffin-embedded tissues, both following the manufacturer’s protocol (Qiagen). RNA Quantity and purity were checked using a Nanodrop ND-1000 Spectrophotometer and Qubit 2.0 Fluorometer (ThermoFisher, Waltham, MA, USA). The nCounter canine IO panel (NanoString, Seattle, WA, USA) was performed per the manufacturer’s instructions. The NanoString gene expression data were analyzed using nSolver software (v4.0). Quality control metrics were analyzed using the NanoTube package; all samples shown in the manuscript were deemed of sufficient quality based on guidelines provided by NanoTube. The gene expression data were normalized using the “normalize” function from the nanoStringNCTools package. All downstream analyses were performed on normalized data. Hierarchical clustering (Euclidean distance, average linkage) was used for all heatmaps. Gene expression was represented by z-scores of normalized data for all heatmaps. Immune cell expression was inferred using the average expression of immune cell marker genes from the safeTME R package, normalized by the average expression of control probes. The RNA quality of P1 and P2 was poor and the samples were excluded from the transcriptomic analyses. 

### 2.8. Statistical Analyses

Linear regression analysis was performed for the evaluation of individual IT-induced changes in tumor size between treatment points, follow-up points, and the start of treatment. To evaluate potential toxic and immunological effects of IT therapy in dogs, a two-tailed Student’s *t*-test or, as appropriate, Wilcoxon test was performed to compare therapy-induced changes in blood cell numbers and biochemistry variables. Two-tailed *p*-values smaller than 0.05 were considered statistically significant. Statistical analyses were carried out using IBM SPSS Statistics program (version v.25; Armonk, NY, USA) and GraphPad Prism (version 7.02; GraphPad San Diego, CA, USA) software. Additional details are described in the Supplemental File. BioRender was used to design the graphical abstract. 

## 3. Results

### 3.1. Clinico-Pathological Characteristics of CMC Patients

Six canine patients were enrolled with characteristics reflecting the heterogeneity seen in human BC patients: middle–advanced age (9 to 13 years old), different breeds, and human-related comorbidities like cholesterolemia, chronic kidney disease, increase in hepatic enzyme activity, and blood variations associated with a chronic and/or inflammatory disease, such as mild anemia, increases in plasmatic globulins and in the number of peripheral blood leukocytes (Table S3), different tumor subtypes (ER, PR, and TN), low (I), intermediate (II), and high histological tumor grade (III), and clinical stage I to V (Table 1).

In addition to the injected target tumor, P2 had three tumors on the left mammary glands (P2.2–P2.4); P4 had two tumors, one on the right (P4.2) and one on the left (P4.3) mammary chains; and P5 had one tumor on the left (P5.3) and one on the right (P5.4) mammary chain; these two tumors were initially tracked, but during treatment, the tumors became too small to correctly measure, and their measurements were discontinued. 

At the time of diagnosis, thoracic radiographs indicated that patient P5 had five lung metastatic nodules (three left, P5.M1–M3, and two right, P5.M4–M5 nodules). Patient P6 previously underwent a mastectomy on the right mammary chain and, at the time of enrollment, she had a primary tumor on the fifth right mammary gland not resected when the mastectomy was performed (this tumor was treated), and also had two left (P6.M1–M2) and one right (P6.M3) lung metastatic nodules.

### 3.2. acPD-1 Monotherapy or acPD-1/CPMV Combined Therapy Is Safe

No skin reactions in the tumor injection site or changes in the physical status of the dogs were observed in the treated dogs during the ~4 h observation period in the veterinary clinic. Per the QOL questionnaire, during the 4-week treatment period, all owners reported all treated dogs being more alert, more active, and some having an increased appetite, and being of good physical status. This status remained the same for the three dogs during the long-term treatment. 

A hemogram analysis indicated no significant changes in leukocytes, lymphocytes, monocytes, neutrophils, and platelet numbers in any dogs. However, a decrease in the erythrocyte numbers, hemoglobin, and hematocrit was observed in P1 and P5 during the first week (D2–D7) compared with the basal levels at D0 (Figure S1A–C). Although these three parameters were below the normal range at the beginning of the combined therapy in P5, at D7, we observed a sustained drop in those three markers. We did not observe any CPMV-related drops in these parameters in previous CMC studies [33,34], and those changes could be related to either the disease in this dog and/or IT acPD-1 therapy. Hence, to avoid potential adverse events, P5 did not receive the planned acPD1 dose (0.25 mg) on D9, but did receive that dose when she was clear to continue IT treatments in the next week. Once P5 continued with the combined therapy, those variables rose slightly, but remained below the normal range up to D113. A similar trend was also observed in P1, but she received the planned acPD-1 up to 1 mg. P5 ended up with only 0.5 mg injected in each of her two tumors (or 1 mg total), instead of the planned 1 mg per tumor or 2 mg total. The changes in blood and biochemistry variables were not of concern in the other treated dogs (Figure S1A–C).

While total serum proteins were high before treatment in most of the dogs, except for P2, there was a decreasing trend toward the normal range during treatment (Figure S2A). Similarly, the total globulin levels were a little higher than the normal range in P1, P3, and P6, but the levels dropped during treatment (Figure S2B). The albumin levels were within the normal range during treatment, with P1 and P5 remaining in the low range or below the normal range during treatment (Figure S2C). The albumin-to-globulin ratio remained below the normal range as it was before treatment, and no significant changes were observed in total proteins or in cholesterol levels during treatment when compared with basal levels. 

For liver enzymes, alanine aminotransferase (ALT) and aspartate aminotransferase (AST) were evaluated. At D0, P1, P5, and P6 had high ALT levels, which decreased to the normal range during treatment (Figure S3A). The AST levels remained in the normal range in most of the dogs, except in P1 and P5, which were low (P1) or below (P5) the normal range at D0 and remained below the normal range during the treatment period (Figure S3B). Kidney function measured by creatinine levels showed slight fluctuations in P3 and P5; it was high in P1 at D0 and remained high during the treatment up to D73 (Figure S3C). The urea levels were high in P1 and P3 and remained higher during the whole treatment; some fluctuations were seen in P5, but remained in the normal range by the end of treatment (Figure S3D). It should be noted that abnormal renal function was also a limitation for surgery in P1 and she died of renal failure ~10 weeks after the last CPMV/acPD-1 ITI treatment.

Collectively, neoadjuvant IT acPD-1 and IT CPMV/acPD-1 therapy is safe without serious irAEs or local reactions requiring medical intervention in treated patients.

### 3.3. acPD-1 or acPD-1/CPMV Therapy Controls Tumor Burden in Injected and Noninjected CMC Tumors

**Figure 2:**
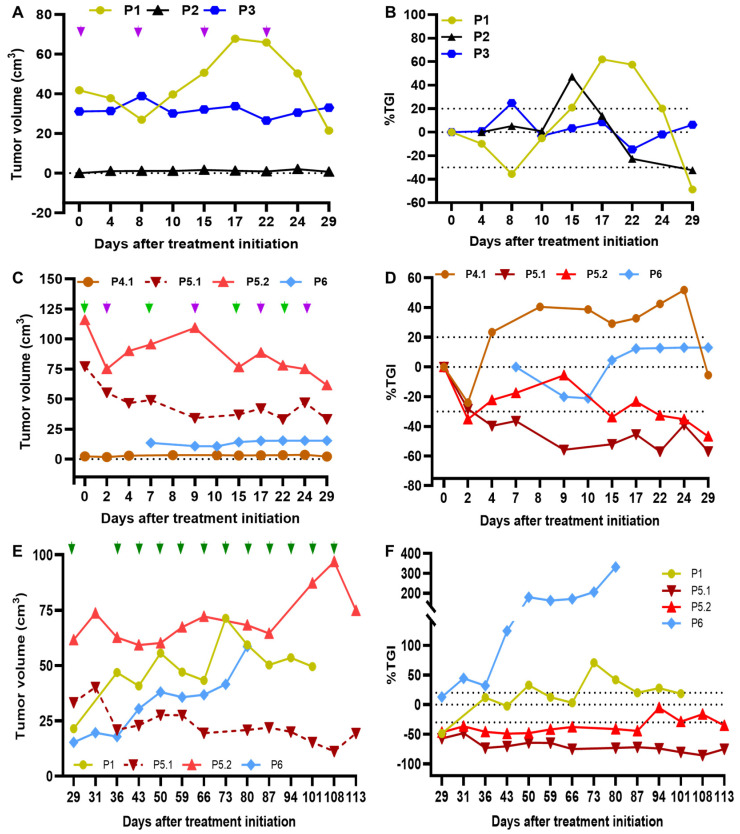
acPD-1 and CPMV/acPD-1 treatments are associated with tumor control. Weekly acPD-1 as monotherapy (patients P1-P3; purple arrows; (**A**)) or CPMV (light green arrows in (**C**)) plus acPD-1 (purple arrows in (**C**)) was administered for four weeks to patients P4-P6; in P5, tumors P5.1 and P5.2 were treated. Starting on D29, long-term weekly IT CPMV/acPD-1 treatment ((**E**); dark green arrows) was administered to target tumors in patients P1, P6, and P5 (P5.1 and P52 were treated). The %TGI (relative to D0) in (**B**,**D**,**F**) indicates the SD in the dotted areas (−30 to 20), above 20% indicates PD, and below −30% indicates a partial response.

During the 4-week treatment period in the IT acPD-1 arm (P1-P3), we observed a tumor reduction in P1 followed by subsequent tumor growth and then tumor reduction, while tumors did not grow in P2 and P3 (Figure 2A). The %TGI indicated that SD was observed in P3 and PR in P1 and P2 by D29 (Figure 2B). 

In the IT CPMV/acPD-1 arm (P4–P6), there was an initial small tumor reduction in both P4 and P6 tumors with subsequent tumor growth and a reduction in P4 but not in P6, where the tumor grew from D10 to D17 and remained controlled up to D29 (Figure 2C). Patient P5 presented with two large tumors in her fourth right (77.3 cm^3^; P5.1) and left (115.7 cm^3^; P5.2) mammary glands, which were treated as individual tumor masses. As illustrated in Figure 2C, a higher tumor reduction was observed in the right tumor than the left tumor up to D29. The %TGI indicates that CPMV/acPD-1 treatment resulted in PR in P5 in both tumors and SD in P4 and P6 (Figure 2D). 

The ORR (CR+PR) during the 4-week treatments was 67% (two PR out of three patients) in acPD-1, and 33% (one PR out of three patients) in CPMV/acPD-1. The CB was 100% for both arms (acPD-1, two PR and one SD; and CPMV/acPD-1, one PR and two SD).

During long-term IT CPMV/acPD-1 treatment, we observed tumor growth in injected tumors in both P1 and P6 starting on D29, with a sharp increase in tumor growth in P6 at D36, while we observed a continued response in P5.1 and, to a lesser extent, in P5.2 (Figure 2E). Regression analysis indicated a significant tumor reduction in the P5.1 tumor (*p* < 0.001), and tumor growth in P1 (*p* = 0.024) and P6 (*p* < 0.001) (Table S4). The %TGI indicated SD in P1, PD in P6, and PR in both P5 tumors (Figure 2F). The ORR was 33% (PR in one out of three patients); the CB was 67% (one PR and one SD out of three patients).

The systemic effect of the IT treatment on noninjected malignant tumors was tracked in patients P2 and P4. P2 had three noninjected tumors in the left mammary chain, with no tumor growth observed in two small tumors (<1.0 cm^3^; P2.2 and P2.3), and tumor reduction was observed in the larger (~3.0 cm^3^, P2.4) tumor. The larger right tumor in P4 (3.5 cm^3^, P4.2) grew, while the smaller left tumor (~0.2 cm^3^, P4.3) did not grow during the four-week treatment period (Figure S4). 

The exploratory itRECIST evaluation indicated that SD was observed in the acPD-1 (Figure S5A) and CPMV/acPD-1 arms (Figure S5B) during the four-week treatment, as well as in the long-term with CPMV/acPD-1 (Figure S5C). While in the noninjected tumors P2.2 and P2.3 we observed SD and PR in P2.4, P4.2 had PD and P4.3 had SD (Figure S5D). The Swimmers plot illustrating the response to CPMV/acPD-1 in target injected lesions in CMC patients based on itRECIST criteria is presented in Figure S6A.

### 3.4. Abscopal Effect of CPMV/acPD-1 Treatments on Established Lung Metastases

**Figure 3:**
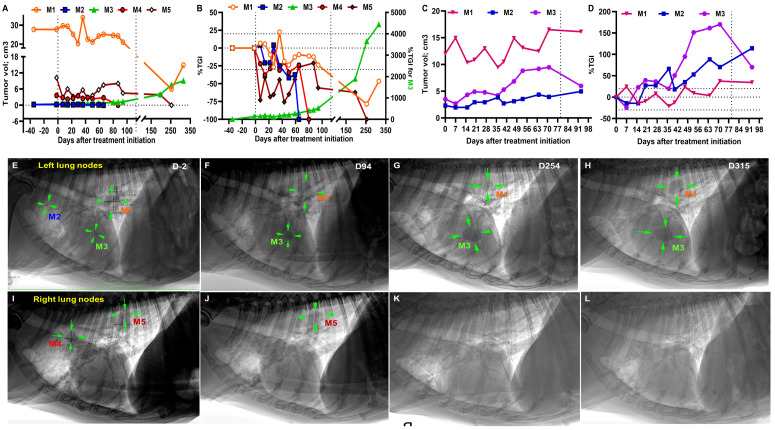
CPMV/acPD-1 treatments had a variable abscopal effect on established lung metastases. Of the five established lung metastatic nodules in P5, M1, M2, M4, and M5, nodules were responsive to CPMV/acPD-1 treatments, while M3 was not and had steady tumor growth (**A**). During treatment, we observed CR in M2 and M4, PR in M1 and M5, and PD in M3 (**B**). After surgery on D113, we observed CR in M5, PR in M1, and PD in M3 (**B**). In P6, M1 and M3 nodules showed transient responses, and M2 slowly grew during treatment and after surgery on D79 (**C**). In this patient, we observed transient SD in the largest M1, and a rapid transition from SD to PD in M2 and M3 (**D**). The vertical dotted line on the x-axis indicates palliative surgery (mastectomy or tumor resection) of the primary tumors. Representative radiographs illustrate tumor changes in the left (**E**–**H**) and right (**I**–**L**) lung metastatic nodules in P5 before (D-2), during (up to D94), and after surgery (D254-D315). Note changes in tumor volume in M1 and M5, as well as the absence of M2, M4, and M5.

We were able to track real-time changes in the lung metastatic nodules with thoracic radiographs during and after treatment in patients P5 and P6. Patient P5 had three left nodules: M1 (27.80 cm^3^), M2 (0.24 cm^3^), and M3 (0.46 cm^3^) and two right nodules: M4 (3.61 cm^3^) and M5 (10.21 cm^3^)(Figure 3A). During treatment, the five metastatic nodules had variable responses to CPMV/acPD-1, with M2 and M4 nodules not being detectable by D65 and D94 (Figure 3A), M1 and M5 were responsive, but M3 was not, and grew steadily up to D315 (A). After surgery on D113, M5 tumor reduction was observed and by D254, it was no longer observed by radiograph, while tumor reduction was seen in M1 (Figure 3A) with subsequent tumor growth observed by D315. In P5, during treatment, we observed CR in M2 and M4, PR in M1 and M5, and PD in M3 (Figure 3B). After surgery, we observed CR in M5, PR in M1, and PD in M3 (Figure 3B). Representative radiographs illustrate the tumor changes in the left (Figure 3E–H) and right (Figure 3I–L) lung nodules in patient P5 before (D-2), during (up to D94), and after treatment surgery on D113 (D254–D315).

In P6, the M1 (12.0 cm^3^) and M3 (3.5 cm^3^) nodules showed transient responses, and M2 (2.3 cm^3^) slowly grew during treatment and after surgery on D79 (Figure 3C). In this patient, we observed SD in the largest M1 nodule and a rapid transition from SD to PD in M2 and M3 (Figure 3D). 

The exploratory itRECIST evaluation for the lung metastases indicates that, during treatment, P5 had CR in M2 and M4, SD in M1, and PD in M3 and M5 (Figure S7A). After treatment, we observed CR in M5, SD in M1, and PD in M3 (Figure S7A). Patient P6 had SD in M1 and M2, and PD in M3 (Figure S7B). The Swimmers plot illustrating the response to IT CPMV/acPD-1 in noninjected distant lung metastases in P5 and P6 patients based on itRECIST evaluation is presented in Figure S6B.

While survival outcomes were not within the objectives of this study, the outcomes for the acPD-1 (P1–P3) and CPMV/acPD-1 (P4–P6) arms were as follows: After four weeks of treatment, P1 was in good health and was treated further. P2, P3, and P4 underwent planned surgery with adjuvant therapy administered to P3. P2 and P4 are alive and in good health (472 days after the last treatment); P3, died of a cardiopathy issue 97 days after their last treatment. During long-term CPMV/acPD-1 treatments, P1 received weekly treatment up to D94, and remained without treatment up to D162, when she died of renal failure (68 days after the last treatment); P6 underwent palliative surgery at D79, followed by adjuvant chemotherapy, and died at D219 of lung metastasis (139 days after their last treatment); P5 received treatments up to D108 and palliative surgery at D113, followed by adjuvant chemotherapy, and was euthanized at D386 (278 days after their last treatment). Overall survival is illustrated in Table 1. 

### 3.5. acPD-1 and CPMV/acPD-1 Treatments Induced Changes in the TME

**Figure 4:**
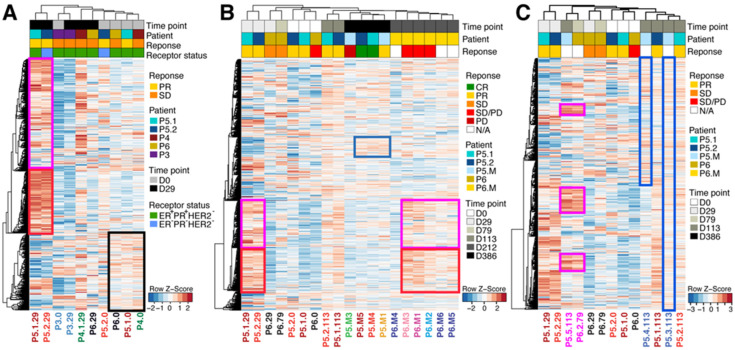
acPD-1 and CPMV/acPD-1 treatments induced transcriptomic changes in the tumor microenvironment. During the four-week treatment period, acPD-1 and CPMV/acPD-1 treatments affected gene expression in injected tumors (**A**). Extended CPMV/acPD-1 treatments affected gene expression in injected tumors, noninjected lung metastases (**B**), and noninjected tumors and inguinal nodes (**C**). Details in text. Legends: Each row indicates a gene; each column, an individual patient. Patient characteristics are indicated on the right side of the graphs. Patients are identified by a number (P3–P6), followed by the number of the treated or untreated tumor or metastatic lesion, and the day the sample was collected. P3 and P6 had a single treated tumor and are represented as P3.0 and P6.0, while P4 and P5 had injected tumors (P4.1, P5.1, and P5.2), noninjected tumors (P5.3.113 and P5.4.113), and inguinal nodes in P5 (P5.5) and P6 (P6.2). The numbers at the end of each sample represent the day when the sample was collected. Metastatic lesions are represented by the letter M. Gene expression levels are represented by z-scores.

Transcriptomic analysis indicated the presence of altered gene expression in injected and noninjected samples. During the four-week treatment course (D0 and D29), hierarchical clustering reveal three gene clusters (Figure 4A; gene list in Table S5). Pathways associated with TLR1 to TLR4 cascades were enriched in clusters 1 and 3 (Figure 4A, pink and black boxes, respectively; and Figure S8), whereas pathways associated with complement, TNF, interleukins, and chemokine pathways were enriched in cluster 2 (Figure 4A, red box; and Figure S8). P5.1 and P5.2 tumors showed a strong upregulation of genes in cluster 2 on D29 compared with all other samples, potentially reflecting a patient-specific response. Genes in cluster 1 seemed to be associated with a treatment response to CPMV/acPD-1, as all D29 tumors treated with CPMV/acPD-1 showed a modest upregulation of genes in this cluster. This may be related to the known effect of CPMV on TLR2, TLR4, and TLR7 [19]. Genes in cluster 3 seemed to be associated with treatment response, as these genes were modestly downregulated in D29 samples. P3, which was treated with acPD-1 only, and had very few altered genes compared with all other samples from both D0 and D29. 

Expanding the hierarchical clustering analysis to samples treated after D29 in both patients P5 and P6, the patients with the largest number of biopsies, we observed a range of changes in gene expression. The two gene clusters (cluster 1 and 2, Figure 4A) upregulated in D29 P5.1 and P5.2 tumors were again reflected in this clustering analysis. Interestingly, genes in these two clusters were also upregulated in metastatic samples in P6 collected on D212 (Figure 4B, pink and red boxes). Genes in P5 noninjected metastases (collected at D386) clustered together, with only a small number of genes showing upregulation in the three responsive nodules (P5.M1, P5.M2.4, and P5.M5), but not in the nonresponsive metastatic nodule (P5.M3; Figure 4B, blue box). Lastly, we noted that genes in clusters 1 and 2 (Figure 4B, red and pink boxes) were lowly expressed in P6 D0 samples and their expression gradually increased over time; P6 D79 showed a slight increase and metastatic P6 D113 showed a clear increase in gene expression.

An abscopal effect on gene expression was also observed in the two P5 untreated tumors we stopped tracking when their tumor growth reduction occurred and measurements became unreliable (P5.3.113 and P5.4.113 in Figure 4C). While gene expression in P5.3.113 (fifth left mammary gland) shared similarities with its treated neighbor P5.2.113 tumor (fourth left mammary gland), gene expression in P5.4.113 (fifth right mammary gland) differed completely from that in the treated P5.1.113 (4th sample from the right) (Figure 4C). Interestingly, the inguinal nodes in P5 (P5.5.113) and P6 (P6.2.79) showed similar gene expression patterns having small common clusters (Figure 4C). 

**Figure 5:**
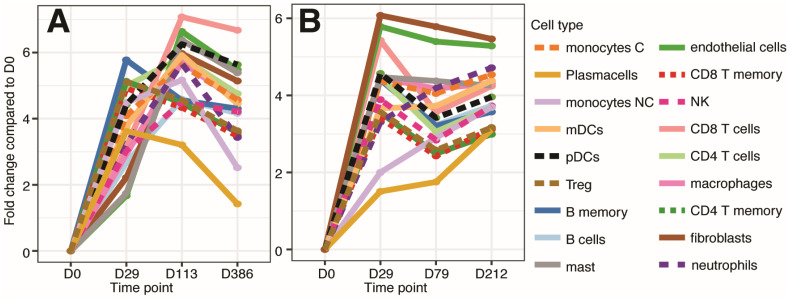
CPMV/acPD-1 treatments induced changes in immune cell contents in injected tumors and noninjected, established metastases. CPMV/acPD-1 treatments increased immune cell content in both P5 (**A**) and P6 (**B**) patients with variations observed in the type of immune cells and the intensity of the increase, with some immune cells having a high content from D0 up to surgery day in P5 (D113), but not in P6 (D79) (comparing CD8 T cells, mast cells, plasmacytoid dendritic cells (pDCs), myeloid dendritic cells (mDC) between P5 and P6) or an increase from D0 to D29 and then a decrease in the content (compare plasma cells between P5 and P6). It is also noticeable that the cell contents from surgery to metastases decreased in P5, while an increase was observed in most of the immune cells in P6 metastases.

Transcriptome analysis of immune cell changes within the TME in CPMV/acPD-1 injected tumors and noninjected metastases showed variable changes in immune cells in both P5 and P6 tumor samples (Figure 5). Most of the immune cell contents increase by D29 in both P5 and P6 tumors. However, CD8 T cells, plasmacytoid and myeloid dendritic cells, neutrophils, monocytes, and B cells remained high, while B memory cells, T reg, CD8 T memory, and plasma cells decreased in P5 by surgery day (D113) (Figure 5A). In the P6 tumor, most of the immune cells decreased by surgery day (D79), except for plasma cells, monocytes, and neutrophils, which increased (Figure 5B). Of note, compared with injected tumors, immune cell contents decreased in P5 lung metastases while most of them increased in P6, except for fibroblasts, endothelial cells, and mast cells, which slightly decreased by D212 (Figure 5A,B). These transcriptional data support the role of CPMV in creating a “hot” tumor microenvironment through the modulation of TLR pathways.

## 4. Discussion

The current use of anti-PD-1 immunotherapy in BC has been minimally effective [4,5]. We have demonstrated good efficacy of IT CPMV in CMC patients [33,34], supporting the potential to implement this safe and effective immunotherapy in CMC, and potentially in human BC. Given the increasing number of PD-1-focused clinical trials in BC [4,35,36], we sought to evaluate the feasibility of combining IT CPMV with IT anti-PD-1 using an optimal animal model, like dogs with spontaneous mammary tumors, to support the implementation of clinical trials of IT CPMV combined with approved anti-PD-1 in human BC patients. While IT anti-PD-1 therapy is expected to be less toxic clinically and economically than systemic anti-PD-1 for humans, systemic anti-PD-1 is commonly used clinically and could be combined with IT CPMV, as many other combinatorial immunotherapies, including IT therapies, are now being performed [4,35,36]. The immunologic rationale for combining IT with systemic anti-PD-1 is that IT injection of an immunostimulatory reagent, such as CPMV, disrupts local immune suppression and expands antitumor effector T cells, which can then improve clinical responses to systemic anti-PD-1.

Two groups have reported clinical studies targeting PD-1 in canine patients [22,47,48,49]. One group reported the safety and efficacy of systemically administered rat–canine-chimeric and caninized anti-PD-1 antibodies in 30 dogs with oral malignant melanoma (OMM) and other spontaneous tumors, including two CMC cases [22,47], and in 37 non-OMM patients [48] and another case report of a canine salivary adenocarcinoma treated with systemic caninized anti-PD-1 [49]. Although most of the dogs previously received various treatments, clinical responses were observed along with irAEs, similar to those observed in humans [24]. 

While some fluctuations in hemogram and biochemistry variables were observed in our CMC-treated patients, none were concerning or suggestive of irAEs requiring medical intervention during the four-week period, or during the long-term combined CPMV/acPD-1 treatments. Hence, our feasibility study demonstrated that neoadjuvant IT acPD-1 whether as monotherapy or combined with IT CPMV is safe, tolerable, and without irAEs, and they could represent a potential treatment option for CMC patients. 

Although the number of CMC patients analyzed was too small to draw strong conclusions, we observed efficacy with both acPD-1 monotherapy and combined CPMV/acPD-1 treatments, including a reduction of tumor burden in the injected and noninjected primary tumors, as well in established lung metastases. We have previously documented that neoadjuvant IT CPMV injections resulted in tumor reduction in both the injected and noninjected tumors (abscopal effect) in CMC patients [33,34]. As IT CPMV was the only therapy provided to CMC patients, the observed systemic response was a bona fide abscopal effect. In agreement with our previous studies, we observed an abscopal effect in noninjected tumors. However, the more striking tumor reduction was observed in noninjected lung metastatic nodules in P5 and P6 patients treated with IT CPMV/acPD-1, suggestive of a potent systemic immune response. The abscopal effect in noninjected tumors and lung node metastases observed manually and with radiographs was confirmed by transcriptomic analysis, which demonstrated the systemic changes in gene expression in (i) noninjected tumors and inguinal nodes with some small gene clusters observed in the samples analyzed; (ii) the three lung metastases, which had tumor changes observed in the radiographs, and three additional lung nodes not seen in P6 radiographs; (iii) variable gene expression in P5 metastases, which differed from the injected and noninjected P5 primary tumors, and P6 metastases; and (iv) a wide range of immune cell activation in injected tumors, noninjected tumors, and metastases as a result of the CPMV/acPD-1-induced systemic response. The decrease in immune cell contents observed in P5 lung metastases compared with an increase in P6 metastases could be related to adjuvant therapy. P5 received toceranib phosphate (a receptor tyrosine kinase inhibitor) [50], while P6 received doxorubicin, which has a positive effect on immune cells [51]. Furthermore, despite being a relapsed mCMC patient, P6 showed responses in the established metastatic nodules. This could suggest that a well-responding patient like P5 may have improved patient outcomes from immune-activating adjuvant therapy, such as doxorubicin, compared to drugs that do not activate the immune system. However, additional studies are warranted to substantiate this observation, and to determine whether specific genes in cluster 2 are associated with the consistent response observed in P5 tumors, but not in other tumors. 

In relation to the abscopal effect on metastases, our findings are significant because once metastatic disease has occurred, there is currently no cure in canine or human patients. Metastatic breast cancer (mBC), whether present at diagnosis (de novo) or occurring later (relapse), is responsible for 90% of BC deaths [52,53]. Of note, women with de novo mBC have superior outcomes compared with women with relapsed mBC [52]. Similarly to human mBC, P5 is a de novo and P6 is a relapse metastatic CMC patient (mCMC), and we observed better responses in the primary tumors and metastatic lung nodules in P5 compared to P6. Adding additional treatments to CPMV/acPD-1 could improve the observed reduction in lung metastases and, therefore, survival outcomes. 

While the abscopal effect observed with the CPMV/acPD-1 in noninjected tumors and, especially, in the lung metastases in the two mCMC patients is remarkable, this study was focused on safety and dosing information. Although striking responses were observed for some treated and untreated tumors, the sample size was too small for statistical validation of efficacy. Because our findings demonstrate that the therapy is safe, well-tolerated, and had an effect on distant metastases, we envision future studies enrolling a larger number of high-risk CMC patients (histopathological tumor grades 2 and 3) who are at risk of developing distant metastases and, therefore, their survival outcome, as in human BC, is poor. We also recognize that additional therapeutic agents like IL-12/IL-2 [27,28], anti-OX40, and TLR3/8 agonist [29] could enhance the observed abscopal effect and further improve patient outcomes, and these or other relevant agents could be added in future studies.

It should be noted that the median survival time for mCMC patients treated with surgery alone or with chemotherapy is generally ~50–200 days [54,55,56,57]. While survival outcomes are out of the scope of this small feasibility study, P5’s overall survival was 386 days (9.1 months after the last treatment) with good QOL. The lack of a strong response in P6 could be related to the fact that the treated tumor was originally not resected when P6 underwent mastectomy a long time before entering the trial, and P6 underwent adjuvant chemotherapy. With time, the small tumor evolved into a more aggressive, chemo-resistant tumor. However, CPMV/acPD-1 resulted in SD in the treated tumor, as well as in controlling lung metastases. Hence, CPMV/acPD-1 could be a good treatment option for de novo and relapse metastatic patients, but studies with a larger number of cases are needed. 

## 5. Conclusions

Collectively, our findings demonstrated that IT acPD-1 alone or in combination with IT CPMV is safe and well-tolerated; acPD-1 was effective in controlling tumor burden in injected and noninjected tumors, and CPMV/acPD-1 was effective against lung metastases. These therapies could be effective for CMC patients, with CPMV/acPD-1 representing a novel therapy against mCMC. Given the striking similarities between CMC and human BC, IT CPMV combined with approved anti-PD-1 therapies could be a novel and effective immunotherapy for mBC. 

## 6. Patents

RJN, PS, JLR, and HAP have submitted a patent application related to the subject matter in this publication. NFS, HAP, and SF are inventors of patents relating to the CPMV intratumoral therapy. 

## Figures and Tables

**Table 1:** Clinicopathologic characteristics of enrolled CMC patients.

Patient	Age, y	Weight, kg	Vol.; cm	Histopath. Type	Clinical Stage	Histo. Grade	Receptor Status	Adj. Ther.	OS, Days
P1	13	28.9	6.3	N/A	N/A	N/A	TN	No	162
P2	11	28.0	1.5	Mixed carcinoma	I	I	ER^+^PR^+^HER2-	No	501 ^¥^
P3	10	5.5	4.2	Comedocarcinoma	IV	II	ER^+^PR^+^HER2-	Yes	133
P4	11	2.1	3.4	Mixed carcinoma	II	I	ER^+^PR^+^HER2-	No	501 ^¥^
P5	11	30.0	6.2; 5.4 ^†^	Mixed carcinoma	V	II	TN ^††^; ER^+^PR^+^	Yes	386
P6	9	7.2	3.5	Mixed carcinoma	V	III	ER^+^PR^+^HER2-	Yes	212

Histopath., histopathological tumor grade; Adj. Ther, adjuvant therapy; N/A, not available; ^†^, right tumor; ^††^, the left tumor is triple-negative (TN), and the right tumor is ER^+^PR^+^. OS, overall survival counted from first treatment day up to 31 May 2024. ^¥^, dogs are alive.

## Data Availability

The data and materials are available from the corresponding author upon reasonable request.

## Supplementary Materials

The following supporting information can be downloaded at: https://www.mdpi.com/article/10.3390/cells13171478/s1, Figure S1. acPD-1 or CPMV/acPD-1 treatments induced transient changes in blood cells. A decrease in erythrocytes (A), hemoglobin (B), and hematocrit (C) was observed in P1 and P5. Dotted areas indicate the normal range values for each variable. Figure S2. acPD-1 or CPMV/acPD-1 treatments induced transient changes in blood biochemistry. A decrease in total proteins were observed in some dogs, and slight hyperglobulinemia and hypoalbuminemia were observed in patients P1 and P5. Dotted areas indicate the normal range values for each variable. Figure S3. acPD-1 or CPMV/acPD-1 treatments induced differential effects on treated patients. High ALT levels decreased with treatment in P1, P5 and P6 and remained normal in other dogs (A). AST levels remained low in P1 and P5 (B); creatinine (C) and urea (D) changes were observed in patients P1, P3, and P5. Dotted areas indicate the normal range values for each variable. Figure S4. acPD-1 or CPMV/acPD-1 treatments induced different effects on noninjected tumors. Tumor reduction was observed in all three noninjected tumors in P2 and tumor control in one noninjected tumor in P4 (P4.3) and no response in another noninjected tumor (P4.2). Figure S5. Tumor changes in target injected and noninjected lesions in CMC patients by itRECIST criteria. Percent change from baseline in diameters of target injected lesions in CMC cases treated with IT acPD-1 as monotherapy (A) or IT CPMV/acPD-1 combined therapy (B) during the first 4 weeks of treatment and during the long-term IT CPMV/acPD-1 treatment (C), and percent change from baseline in diameters of noninjected lesions in P2 and P4 patients (D). The broken lines indicate SD (-30 to 20); PD, progressive disease; PR, partial response. Figure S6. Swimmer plots illustrating the response to therapy in target injected lesions and established lung metastases in CMC patients based on itRECIST criteria. (A) Response to the IT treatment during follow-up in the target injected lesions in CMC cases treated with acPD-1 IT as monotherapy (blue lanes) or IT CPMV/acPD-1 combined therapy (green lanes); note that P1 received acPD-1 monotherapy until D29, and then continued with the combined therapy. Note, death of P5 is presented at D250 (*), though she died at D386 (A and B). (B) The abscopal effect of IT CPMV/acPD-1 treatment on established lung metastases in P5 (gray) and P6 (light orange) patients. *Per itRECIST guidelines, unconfirmed PD means the presence of PD, followed by SD, PR, or CR; two consecutive evaluations showing PD are required for a confirmed PD. Figure S7. Tumor changes in non-injected lung metastases in patients P5 and P6 by itRECIST criteria. Percent change from baseline in diameters of noninjected lung lesions in P5 and P6. Surgery was performed at D113 (A) and 79 (B). SD, stable disease (-30% to 20%), PR, partial response (<-30%), PD, progressive disease (>20%), and complete response (-100%). Vertical dotted lines indicate the start of therapy (D0) and surgery day (D113 in A and D79 in B). Figure S8. CPMV/acPD-1 treatment had a variable effect on the reactome pathways in injected tumors. The top 10 immune-related reactome pathways are highlighted for clusters 1 (A), 2 (B), and 3 (C). Table S1. Breed and spayed status of enrolled CMC patients. Table S2. List of primary antibodies used for immunohistochemistry. Table S3. Hemogram and biochemistry results of CMC patients before treatment. Table S4. Regression analysis of tumor growth. Table S5. List of genes identified in clustering analysis.

## List of Abbreviations

ALT: alanine aminotransferase; AST: aspartate aminotransferase; BC: breast cancer; CB: clinical benefit; cm^3^: cubic centimeters; CR: complete response; CPMV: cowpea mosaic virus; IHC: immunohistochemistry; irAEs: immune-related adverse events; IT: intratumoral; itRECIST: Response Criteria for Intratumoral Immunotherapy in Solid Tumors; ORR: overall response rate; PD-1: programmed cell death 1; PR: partial response; PD: progressive disease; QOL: quality of life; SD: stable disease; (%) TGI: percentage of tumor growth inhibition; TME: tumor microenvironment; Treg: regulatory T cells; Tv: tumor volume.
