# Supplementary material for: An Abscopal Effect on Lung Metastases in Canine Mammary Cancer Patients Induced by Neoadjuvant Intratumoral Immunotherapy with Cowpea Mosaic Virus Nanoparticles and Anti-Canine PD-1
Source: Cells. 2024 Sep 3;13(17):1478. doi: 10.3390/cells13171478 (PMC11394642; doi:10.3390/cells13171478)
Supplement: Supplementary file 1 [file cells-13-01478-s001.zip › cells-3121134-supplementary.pdf]

Supplemental file

Canine patient recruitment and selection criteria

Female companion patients diagnosed with mammary cancer with a tumor of at least 1.5 cm were recruited based on a direct correlation between the malignant nature of mammary tumors and the size with tumor smaller than this defined size could result in enrolling patients with benign tumors [1,2]. Inclusion criteria at diagnosis included absence of severe infection or ulceration of the mammary target tumor; absence of chronic life-threatening disease or any systemic disease that could influence the immune system response (such as endocrinopathies, immune-mediated disease, leishmaniasis and ehrlichiosis); no treatment with immunosuppressive drugs. Dogs with clinical signs compatible with inflammatory mammary carcinoma (as pain, warmth, erythema) were excluded. The use of concurrent continuous treatments with non-steroidal drugs and without apparent therapeutic effects before study inclusion was allowed. For this trial, we followed the ARRIVE guidelines 2.0 [3] (supplemental file 1), and, whenever possible, we followed the guidelines from the "Human Intratumoral Immunotherapy Expert Recommendations" [4].

Hematological and biochemical analyses.

A blood sample (~10 ml) was collected from each patient at D0, and weekly thereafter before any treatment to evaluate hematologic and biochemistry changes induced by IT treatments. The hemogram includes the analysis of erythrocytes, hemoglobin, hematocrit, mean corpuscular volume, mean corpuscular hemoglobin, mean concentration of corpuscular hemoglobin, coefficient of variation of erythrocyte distribution, the number of total leukocytes, neutrophils, monocytes, lymphocytes, and platelets, mean platelet volume, platelet volume distribution, and total proteins. Hematological analyses were performed using a standard hematology analyzer (BC-30 Vet, Mindray Animal Care, Shenzhen, China). The biochemistry assays included renal markers (urea, creatinine), hepatic transaminases (alanine aminotransferase (ALT) and aspartate aminotransferase (AST)), total proteins (albumin, globulin, albumin/globulin ratio), and cholesterol (Table S2). The biochemical panel, total protein, and albumin of each patient was performed using reflection spectrophotometry (CM 250 Wiener Lab, Rosario, Argentina).

Concomitant medications standard adjuvant therapy

CMC patients were treated with concomitant medications at the discretion of the attending veterinarian. These included gastric protectants prescribed intermittently including omeprazole; various non-steroidal anti-inflammatory medications (carprofen, gabapentin, cannabidiol) were prescribed on alternating schedule while on study. As adjuvant chemotherapy, P3 received chlorambucil (4 mg/m^2) and previcox (5 mg/kg); P5 received palladia (2.5 mg/kg); chlorambucil (4 mg/m^2), previcox (5 mg/kg); and P6 received doxorubicin (1 mg/kg).

Surgical procedures

All fine needle aspiration were collected under sedation. One to two weeks after the last immunotherapy treatment, three dogs were surgically treated as follows: regional mastectomy was performed when the largest diameter of the injected tumor was smaller than 3 or 5 cm in dogs with a weight lower or greater than 10 kg, respectively, and complete unilateral mastectomy when it was higher than 3 or 5 cm (if dog´s weight was lower or greater than 10 kg, respectively) or if

multiple nodules were present along the mammary chain. If nodules were present in both mammary chains, patients initially underwent mastectomy of the chain containing the injected target tumor and, after 3-4 weeks, a second regional/complete mastectomy was performed. One dog underwent a second mastectomy. If the patient was not spayed at diagnosis, ovariohysterectomy was performed at the time of the first mastectomy. For surgical procedures, all patients received medetomidine and methadone (10 and 300 micrograms/kg, intramuscularly, respectively), followed by induction with propofol (1 mg/kg, intravenously) and inhalational anesthesia with isoflurane (1.5%-2.5%). Intravenous cephazolin was given 20 min before surgery (22 mg/kg). Further, depending on the mastectomy procedure, transversus abdominis plane block with bupivacaine (up to 2 mg/kg) and/or epidural anesthesia using morphine (0.1 mg/kg) plus bupivacaine (up to 2 mg/kg) was provided. Diffusion catheters were placed during surgery in order to administer bupivacaine (1-2 mg/kg every 6h) in the post-operative period. Catheters were left in place for 3 days. Soft sterile wound dressings and a tubular mesh were placed to cover the wound. Post-surgical therapy also included firocoxib (5 mg/kg, orally every 12 h for 7 days) and tramadol (3 mg/kg, orally every 12 h for 3 days). No post-operative antibiotics were prescribed. The wounds healed uneventfully, and skin sutures were removed after 12 days.

Immunohistochemistry (IHC) assays

Single 4 μm tumor tissue sections were used for histopathology and IHC. Slides were baked at 60 degrees Celsius for 30 minutes prior to being loaded on the Leica Bond Rx autostainer (Leica Biosystems Inc.). Automated protocol includes paraffin dewax using Leica Bond Dewax Solution (Leica Catalog# AR9222) and antigen retrieval using Bond Epitope Retrieval Solution 2 (Leica Catalog# AR9640) incubated for 20 minutes at 100 degrees Celsius. Following antigen retrieval and blocking for endogenous peroxidase, a protein block of horse serum and Bovine serum albumin (1.3%) was applied and incubated at room temperature for 60 minutes. The dilution, incubation time, and source of primary antibodies are described in table S3. Following primary antibody incubation, Biocare's Rabbit on Canine HRP polymer (Biocare Catalog# RC542) was applied for 40 minutes at ambient temperature. Visual detection is completed using Leica Bond Refine Detection System (Leica DS9800) with DAB chromogen and hematoxylin counterstain. Following completion on the Leica Bond Rx, slides are briefly washed in distilled water then dipped through 3 changes of 100% ethanol into 4 changes of xylene. From xylene, slides are cover slipped using Sakura Tissue-Tek Glass Mounting Medium (Sakura Finetek, Catalog# 6419). The same positive tissue control was used as the negative control slide without the primary antibody.

Scoring of IHC markers

The estrogen receptor (ER), progesterone receptor (PR) and HER2 receptor status was defined per guidelines described elsewhere [5]. Biomarkers are reported following REMARK guidelines [6].

Statistical analyses

Primary outcomes were biosafety, measured by evaluation of hematological and biochemistry changes in blood and plasma. Secondary endpoints included ORR, irAEs, QOL, efficacy, measured by tumor volume reduction of the injected target, noninjected tumors, and lung metastatic nodules. For evaluation of individual therapy-induced changes in tumor size between treatment points and follow-up points and start of treatment, linear regression analysis was

performed. To evaluate potential toxic and immunological effects of IT treatments in dogs, a two-tailed Student's t-test or, as appropriate, Wilcoxon test were performed to compare treatment-induced changes in blood cell numbers, plasma levels of total proteins (albumin and globulins), glucose, urea, creatinine, and ALT, and cytokine levels in samples collected before, during treatment, at surgery, and 1 month after surgical procedure. Pearson's correlation coefficient was used to evaluate the correlation between continuous variables. Two-tailed P values less than 0.05 were considered statistically significant. Statistical analyses were carried out using IBM SPSS Statistics program (version v.25; Armonk, NY, USA) and GraphPad Prism (version 7.02; GraphPad San Diego, CA, USA) software.

References:

1. Burrai GP, Gabrieli A, Moccia V, Zappulli V, Porcellato I, Brachelente C, Pirino S, Polinas M, Antuofermo E (2020) A Statistical Analysis of Risk Factors and Biological Behavior in Canine Mammary Tumors: A Multicenter Study. Animals (Basel) 10 (9). doi:10.3390/ani10091687

2. Sorenmo KU, Kristiansen VM, Cofone MA, Shofer FS, Breen AM, Langeland M, Mongil CM, Grondahl AM, Teige J, Goldschmidt MH (2009) Canine mammary gland tumours; a histological continuum from benign to malignant; clinical and histopathological evidence. Vet Comp Oncol 7 (3):162-172. doi:10.1111/j.1476-5829.2009.00184.x

3. Percie du Sert N, Hurst V, Ahluwalia A, Alam S, Avey MT, Baker M, Browne WJ, Clark A, Cuthill IC, Dirnagl U, Emerson M, Garner P, Holgate ST, Howells DW, Karp NA, Lazic SE, Lidster K, MacCallum CJ, Macleod M, Pearl EJ, Petersen OH, Rawle F, Reynolds P, Rooney K, Sena ES, Silberberg SD, Steckler T, Würbel H (2020) The ARRIVE guidelines 2.0: Updated guidelines for reporting animal research. PLOS Biology 18 (7):e3000410. doi:10.1371/journal.pbio.3000410

4. Luke JJ, Davar D, Andtbacka RH, Bhardwaj N, Brody JD, Chesney J, Coffin R, Baere Td, Gruijl TDd, Fury M, Goldmacher G, Harrington KJ, Kaufman H, Kelly CM, Khilnani AD, Liu K, Loi S, Long GV, Melero I, Middleton M, Neyns B, Pinato DJ, Sheth RA, Solomon SB, Szapary P, Marabelle A (2024) Society for Immunotherapy of Cancer (SITC) recommendations on intratumoral immunotherapy clinical trials (IICT): from premalignant to metastatic disease. J ImmunoTher Cancer 12 (4):e008378. doi:10.1136/jitc-2023-008378

5. Peña L, Gama A, Goldschmidt MH, Abadie J, Benazzi C, Castagnaro M, Díez L, Gärtner F, Hellmén E, Kiupel M, Millán Y, Miller MA, Nguyen F, Poli A, Sarli G, Zappulli V, de las Mulas JM (2014) Canine mammary tumors: a review and consensus of standard guidelines on epithelial and myoepithelial phenotype markers, HER2, and hormone receptor assessment using immunohistochemistry. Vet Pathol 51 (1):127-145. doi:10.1177/0300985813509388

6. Sauerbrei W, Taube SE, McShane LM, Cavenagh MM, Altman DG (2018) Reporting Recommendations for Tumor Marker Prognostic Studies (REMARK): An Abridged Explanation and Elaboration. J Natl Cancer Inst 110 (8):803-811. doi:10.1093/jnci/djy088

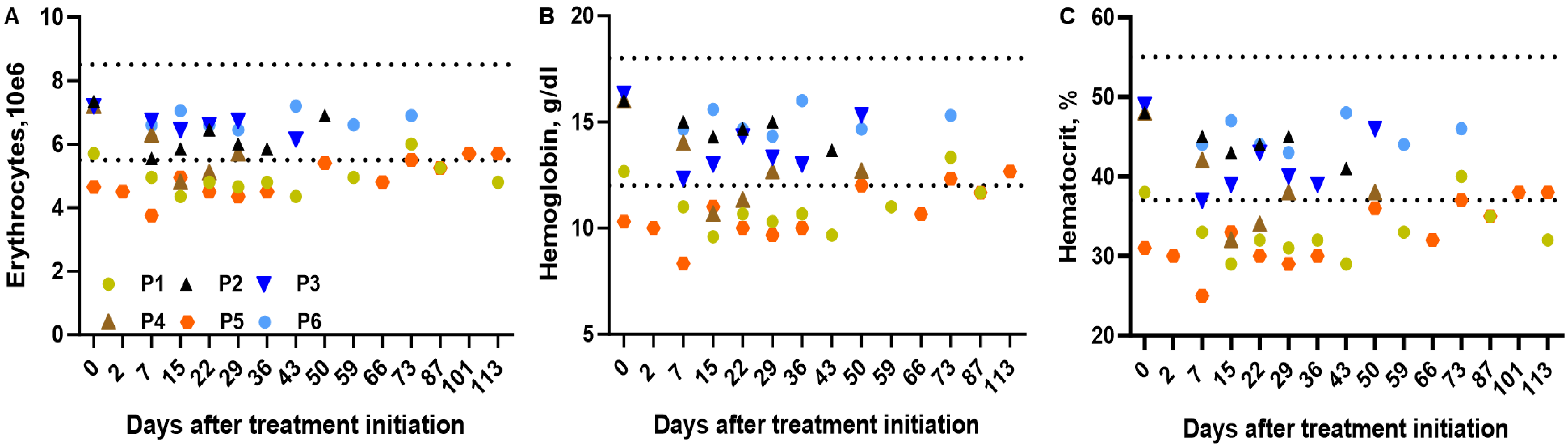

Figure S1. acPD-1 or CPMV/acPD-1 treatments induced transient changes in blood cells. A decreased in erythrocytes (A), hemoglobin (B), and hematocrit (C) was observed in P1 and P5. Dotted areas indicate the normal range values for each variable. Data for the acPD-1 arm (P1-P3) and combined CPMV/acPD-1 (P4-P6) are presented together with the long CPMV/acPD-1 treatment for P1, P5, and P6 patients.

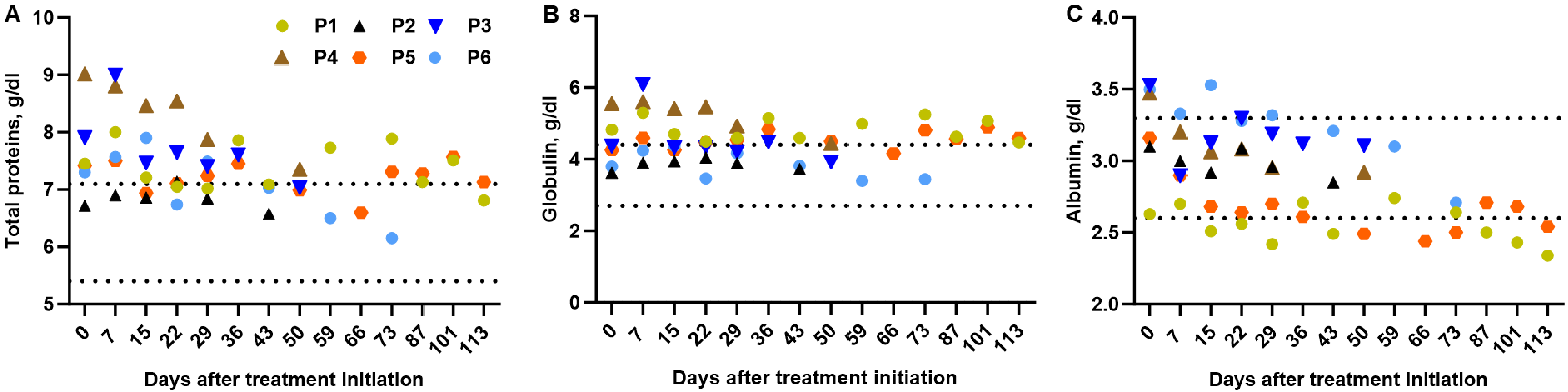

Figure S2. acPD-1 or CPMV/acPD-1 treatments induced transient changes in blood biochemistry. A decrease in total proteins were observed in some dogs, and slight hyperglobulinemia and hypoalbuminemia was observed in P1 and P5 patients. Dotted areas indicate the normal range values for each variable.

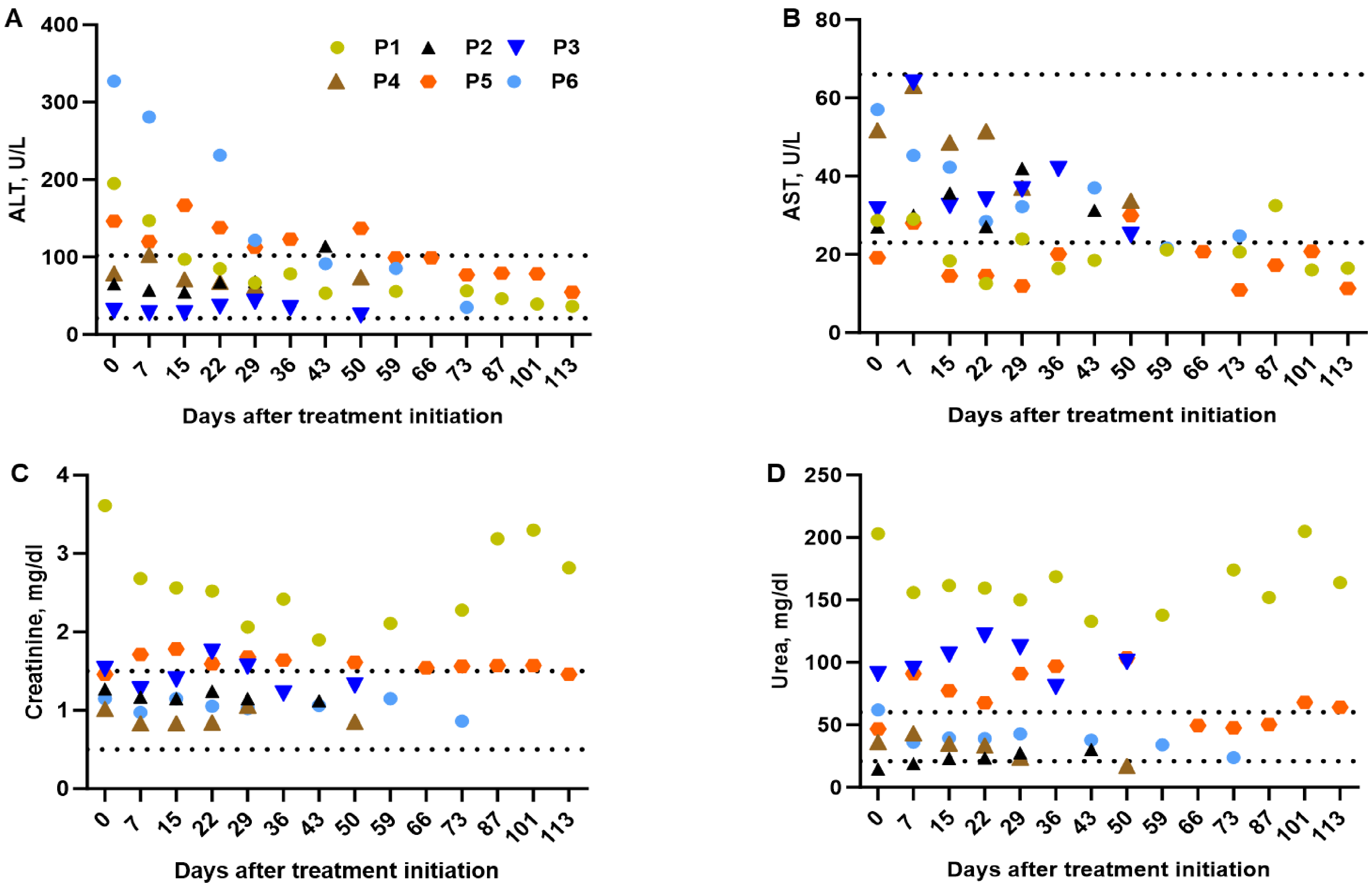

Figure S3. acPD-1 or CPMV/acPD-1 treatments induced differential effects on treated patients. High ALT levels decreased with treatment in P1, P5 and P6 and remain normal in other dogs (A). AST levels remained low in P1 and P5 (B); creatinine (C) and Urea (D) changes were observed in P1, P3 and P5 patients. Dotted areas indicate the normal range values for each variable.

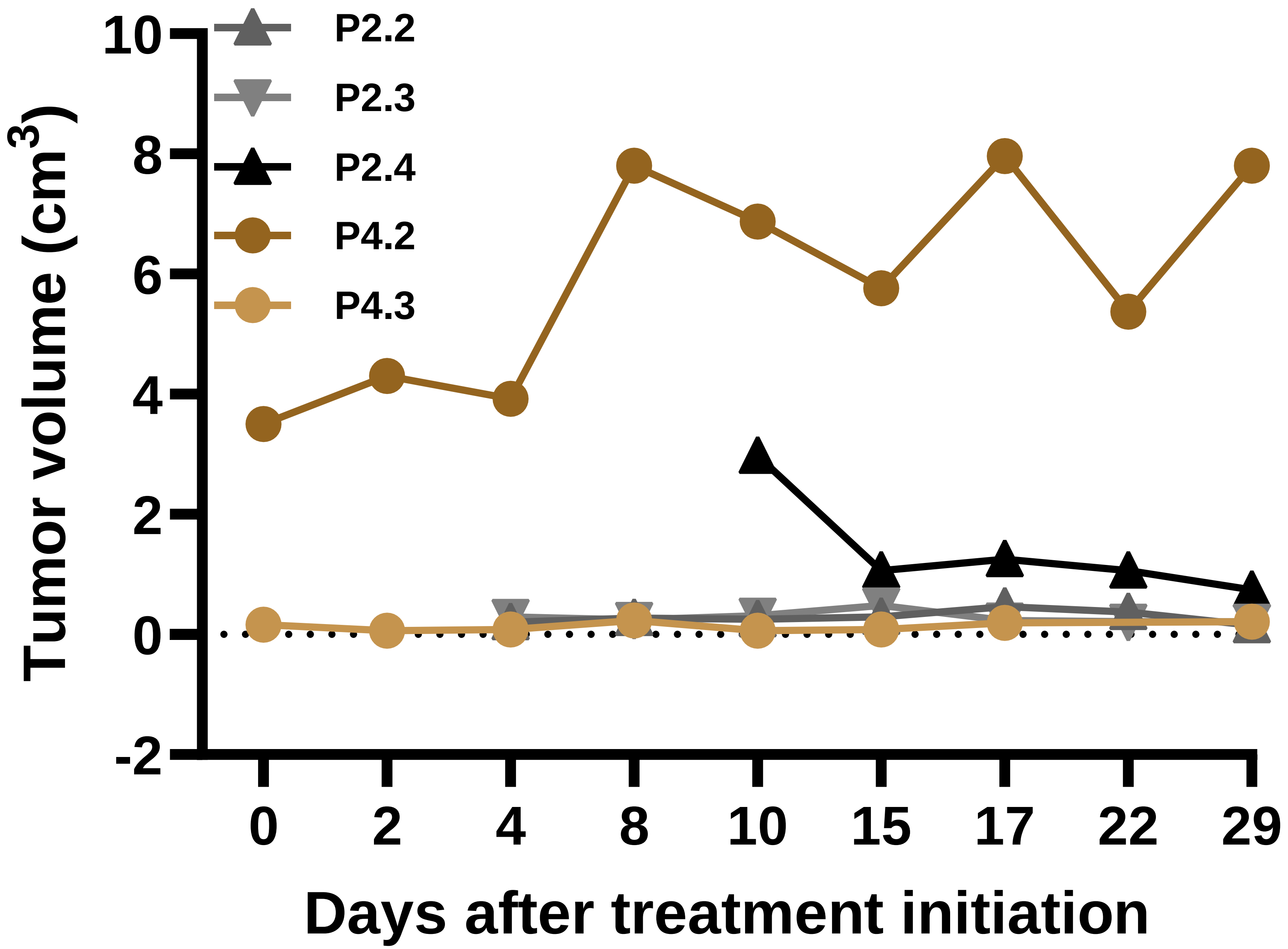

Figure S4. acPD-1 or CPMV/acPD-1 treatments induced different effects on noninjected tumors. Tumor reduction was observed in all three noninjected tumors in P2 and tumor control in one noninjected tumor in P4 (P4.3) and no response in another noninjected tumor (P4.2).

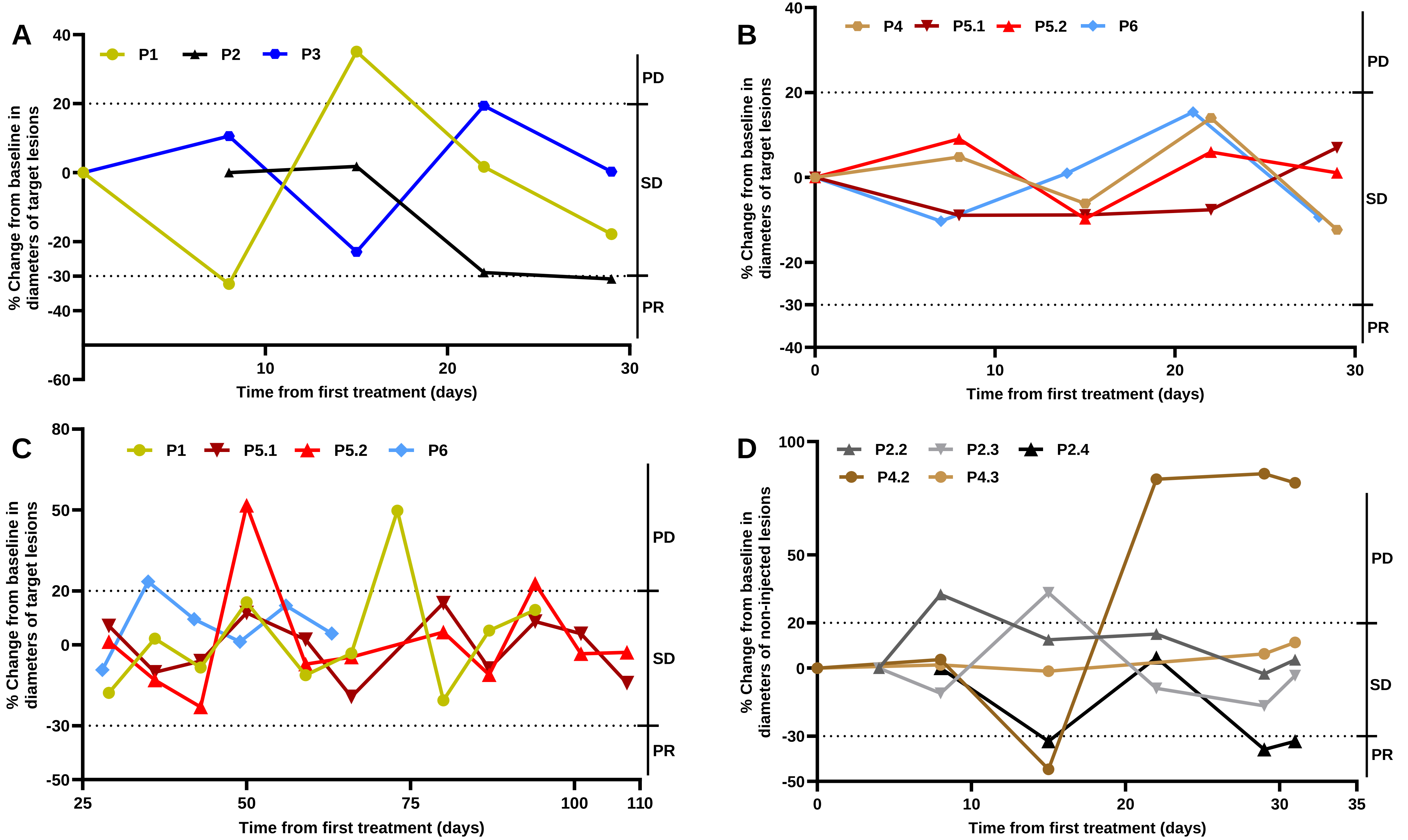

Figure S5. Tumor changes in target injected and noninjected lesions in CMC patients by itRECIST criteria. Percent change from baseline in diameters of target injected lesions in CMC cases treated with IT acPD-1 as monotherapy (A) or IT CPMV/acPD-1 combined therapy (B) during the first 4 weeks of treatment, and during the long-term IT CPMV/acPD-1 treatment (C), and percent change from baseline in diameters of noninjected lesions in P2 and P4 patients (D). The broken lines indicated SD (-30 to 20); PD, progressive disease; PR, partial response.

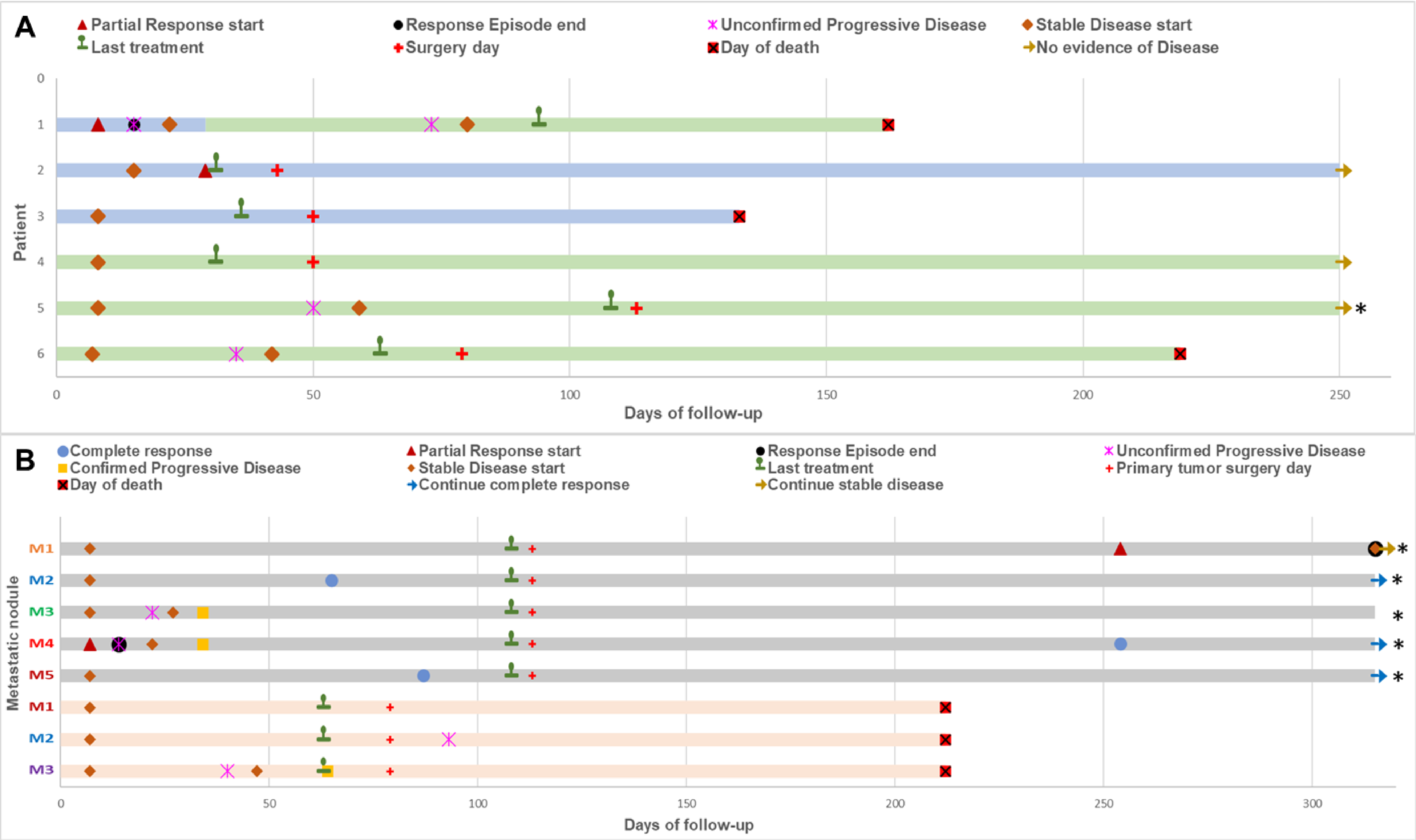

Figure S6. Swimmer Plots illustrate the response to therapy in target injected lesions and established lung metastases in CMC patients based on itRECIST criteria. (A) Response to the IT treatment during follow-up in the target injected lesions in CMC cases treated with acPD-1 IT as monotherapy (blue lanes) or IT CPMV/acPD-1 combined therapy (green lanes); note that P1 received acPD-1 monotherapy until D29, and then continued with the combined therapy. Note, death of P5 is presented at D250 (*) though she died at D386 (A and B). (B) The abscopal effect of IT CPMV/acPD-1 treatment on established lung metastases in P5 (gray) and P6 (light orange) patients. *Per itRECIST guidelines, unconfirmed PD means the presence of PD, followed by SD, PR or CR; two consecutive evaluations showing PD are required for a confirmed PD.

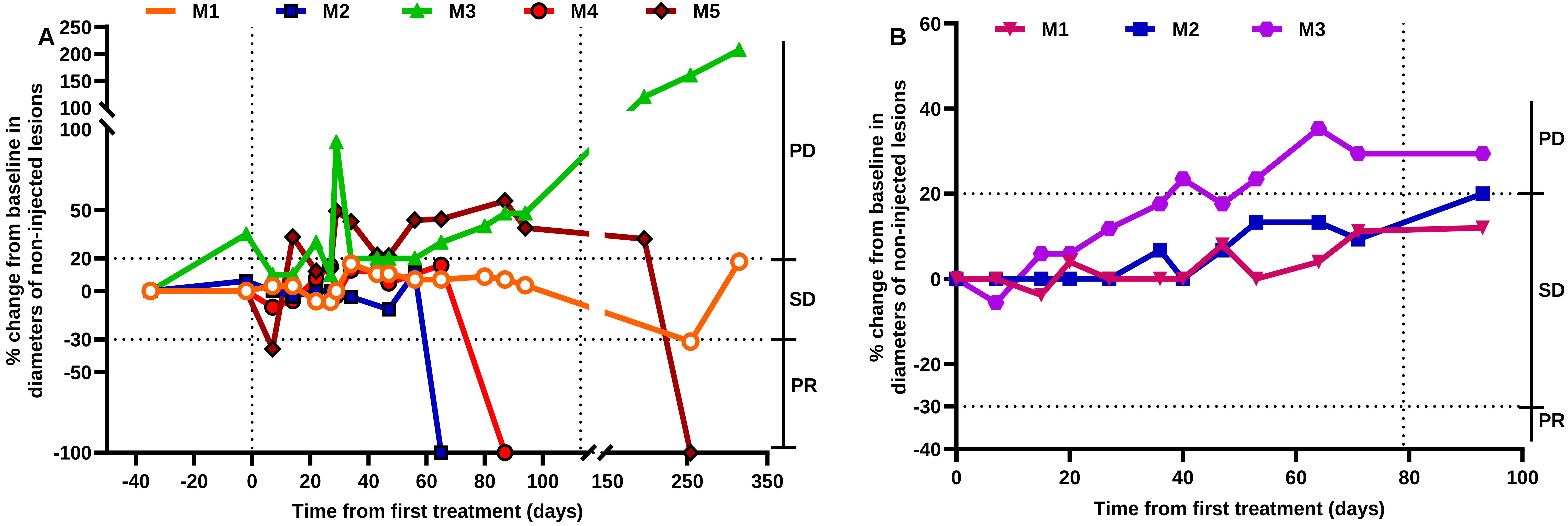

Figure S7. Tumor changes in non-injected lung metastases in patients P5 and P6 by itRECIST criteria. Percent change from baseline in diameters of noninjected lung lesions in P5 and P6. Surgery was performed at D113 (A) and 79 (B). SD, stable disease (-30% to 20%), PR, partial response (<-30%), PD, progressive disease (>20%), and complete response (-100%). Vertical dotted lines indicate the start of therapy (D0) and surgery day (D113 in A and D79 in B).

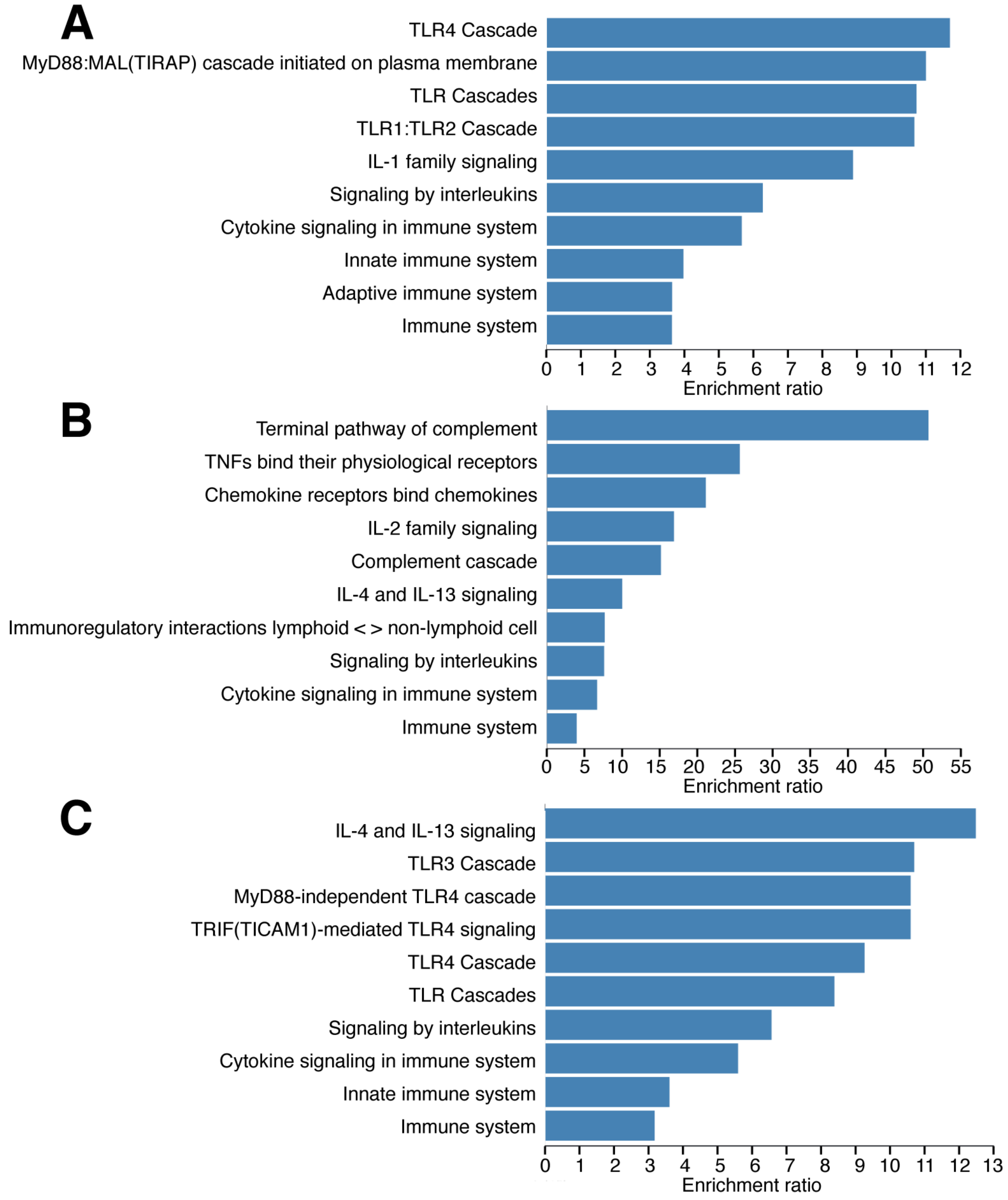

Figure S8. CPMV/acPD-1 treatment has a variable effect on the reactome pathways in injected tumors. The top 10 immune-related reactome pathways are highlighted for clusters 1 (A), 2 (B), and 3 (C).

Table S1. Breed and spayed status of enrolled CMC patients.

Patient	Age, y.	Breed	Spayed
P1	13	Labrador	No
P2	11	Boxer	Yes
P3	10	French poodle mixed with Maltese	No
P4	11	York shire terrier	No*
P5	11	Boxer	No*
P6	9	French poodle mixed with Maltese	Yes

No*, ovariectomy was performed on surgery day.

Table S2. List of primary antibodies used for immunohistochemistry.

Antibody	Clone	Antigen Retrieval	Incubation	Concentration	Provider; cat. #
ER	EP1	BERS2, 96ºC, 20 min	60 min, RT.	RTU	Dako; Cat# IR084
PR	1E2	BERS2, 96ºC, 20 min	60 min, RT.	RTU	Ventana Roche™; 790-2223
HER2	K.929.9	BERS2, 96ºC, 20 min	30 min, RT.	0.6 µg/ml	ThermoFisher Sci., MA5-15050 // 1:100

Legends: ER, Estrogen Receptor; PR, Progesterone Receptor; HER2, human epidermal growth factor receptor 2; RT, room temperature; BERS2, Bond Epitope Retrieval Solution 2.

Table S3. Hemogram and biochemistry results of CMC patients before treatment.

Variable	Normal range	Units	P1	P2	P3	P4	P5	P6
Eritrocytes	5.5-8.5	10e6	5.7	7.2	7.4	7.2	**4.7**	6.6
Hemoglobin	12-18	g/DL	12.7	16.0	16.3	16.0	**10.3**	14.7
Hematocrit	37-55	%	38.0	48.0	49.0	48.0	**31.0**	44.0
Total leukocytes	6-17	10e9/L	8.0	8.0	9.2	8.1	13.3	7.6
Neutrophils	3-11.5	10e9/L	6.0	4.7	5.9	3.9	9.2	6.2
Monocytes	0.15-1.35	10e9/L	0.4	0.8	0.8	0.8	**1.4**	0.5
Lymphocytes	1-4.8	10e9/L	1.6	2.5	2.5	3.4	2.7	**0.9**
Platelets	160-500	10e9/L	179.0	203.0	365.0	395.0	264.0	255.0
Total proteins	5.4-7.1	g/dL	**7.5**	6.7	**7.9**	**9.0**	**7.4**	**7.6**
Albumin	2.6-3.3	g/dL	**2.6**	3.1	**3.5**	**3.5**	3.2	3.3
Globulin	2.7-4.4	g/dL	**4.8**	3.6	4.4	**5.5**	4.3	4.2
Alb/Glob ratio	0.9-2.7		**0.6**	0.9	**0.8**	**0.6**	**0.7**	**0.8**
ALT	21-102	U/L	**195.2**	65.5	31.3	78.5	**146.6**	**280.9**
AST	23-66	U/L	28.7	27.0	31.6	51.7	**19.2**	45.3
Urea	21-60	mg/dL	**203.0**	**14.7**	**90.8**	35.9	46.7	36.2
Creatinine	0.5-1.5	mg/dL	**3.6**	1.3	**1.5**	1.0	**1.5**	1.0
Cholesterol	135-270	mg/dL	256.2	**382.3**	**290.9**	216.1	**373.9**	176.4

Values higher than the normal range are in bold red, and lower, in bold blue. Note that most of the high values decreased with the ITI treatment (See figure S1-3). ALT, alanine aminotransferase; AST, aspartate aminotransferase.

Table S4. Regression analysis of tumor growth.				
**Day**	**P1**	**P5.1**	**P5.2**	**P6**
	Volume	Volume	Volume	Volume
0	41.83	77.28	115.74	
2		55.41	75.00	
4	37.75	46.61	89.97	
7		49.17	95.53	13.56
8	26.93			
9		34.15	109.26	10.83
10	39.72			10.70
15	50.63	37.02	76.54	14.18
17	67.77	42.23	88.61	15.23
22	65.88	33.21	77.90	15.28
24	50.27	47.14	75.00	15.33
29	21.46	33.27	61.59	15.33
31		40.21	73.72	19.60
36	46.88	20.97	62.71	17.91
43	40.83	22.98	59.25	30.46
50	55.60	27.63	60.18	38.01
59	47.06	27.54	67.37	35.68
66	43.27	19.48	72.17	36.83
73	71.40			41.51
80	59.40	20.91	68.33	58.63
87	50.25	21.99	64.54	
94	53.55	20.07		
101	49.58	15.32	87.27	
108		11.20	96.95	
113		19.34	74.84	
123	79.61			
P-value	**0.024**	**<0,001**	0.215	**<0,001**

Tumor measurements up to the last day of follow-up. Red color indicates an increase and blue a decrease in tumor growth.

Table S5. List of genes identified in clustering analysis.

Gene Cluster 1	Gene Cluster 2	Gene Cluster 3
ADGRE5	ABCB1	ABCF1
AKT3	ACOD1	ABL1
ANKRD22	AIRE	ACVR1
APC	AMBP	ADA
AR	ARG1	ADAMTS2
ARG2	ATG12	AKT1
ATG7	BATF	ALCAM
BAX	BLK	ANXA1
BCL10	BTLA	APOE
BCL6	C4BPA	APP
BLNK	C5	ATF1
BRAF	C7	ATF2
BRCA1	C8A	ATG16L1
BRCA2	C8B	ATG5
BTK	C8G	ATM
C1R	C9	ATRX
C2	CAMP	AXL
C3	CARD11	B2M
C3AR1	CCL1	BCL2
C6	CCL16	BCL2L1
CALHM6	CCL17	BCR
CARD9	CCL19	BHLHE40
CASP10	CCL20	BID
CASP8	CCL21	BIRC5
CCL14	CCL22	BMI1
CCL2	CCL24	BST2
CCL7	CCL25	C1QA
CCL8	CCL26	C1QB
CCR5	CCL27	C1QBP
CD14	CCL4	C1S
CD163	CCR10	CASP3
CD180	CCR3	CCL23
CD2	CCR4	CCL5
CD200	CCR9	CCND1
CD209	CD160	CCND2
CD276	CD19	CCR1
CD34	CD1A6	CD164
CD36	CD1B	CD4
CD37	CD1C	CD44

CD38	CD1D	CD47
CD3E	CD1E	CD59
CD40	CD207	CD63
CD48	CD244	CD68
CD53	CD247	CD74
CD55	CD27	CD81
CD58	CD3EAP	CD8B
CD79A	CD40LG	CD9
CD80	CD7	CD99
CD83	CD70	CDH1
CD84	CD79B	CDK1
CD86	CD96	CDK4
CD8A	CDH5	CDKN1A
CFB	CKLF	CDKN2C
CFD	CLEC5A	CEACAM1
CFI	CMA1	CEBPA
CH25H	CPA3	CEBPB
CHEK2	CRP	CLDN1
CHUK	CSF2	CLEC7A
CIITA	CSF3	CLU
CMKLR1	CTSG	COL1A1
CNP	CTSW	COL3A1
COLEC12	CXCL11	CREB1
CR1L	CXCR3	CREBBP
CREB5	DEFB1	CSF1
CSF2RB	DOK2	CSF1R
CSF3R	EBI3	CTSH
CX3CL1	ELANE	CTSS
CX3CR1	EOMES	CXCL14
CXCL10	ESR2	CXCL16
CXCL12	FABP7	CXCL8
CXCR2	FCAR	CYLD
CXCR4	FCER1A	DDR1
CXCR5	FCRL2	DDX5
CYBB	FOXA1	DLA−12
CYFIP2	FOXP3	DLA−64
DDX58	GBP5	DLA−79
DLA−DMA	GH1	DLA−DQA1
DLA−DMB	GZMH	DLA−DQB1
DMBT1	HAMP	DLA−DRA
DNAJC14	HAVCR2	DLA88

DNMT3A	HCST	DUSP1
DOCK2	HDC	DUSP6
DOK1	HNF1A	EGR1
EGFR	IDO2	EP300
ENTPD1	IFNB1	EPCAM
EPSTI1	IFNG	ERBB2
ERBB3	IL11	ERCC3
ERBB4	IL12A	ESR1
ETS1	IL12B	FAS
F13A1	IL12RB1	FAT1
FADD	IL12RB2	FCER1G
FBXW11	IL13	FGFR1
FCGR1A	IL16	FN1
FGFR3	IL17A	FOS
FYN	IL17F	GATA3
G6PD	IL19	GPI
GZMK	IL1B	GSK3A
HBEGF	IL1RL2	GSK3B
HCK	IL2	GUSB
HRAS	IL21	HIF1A
HSD11B1	IL22	HLA−DRB1
ICAM1	IL22RA1	HMGB1
ICAM2	IL22RA2	HMGCR
ICAM3	IL23A	IDH1
IFGGC1	IL23R	IDH2
IFI35	IL24	IFNAR1
IFIH1	IL25	IFNGR1
IFIT2	IL26	IGF1R
IFNAR2	IL27	IGF2R
IGHM	IL29L	IGHG
IKBKG	IL2RA	IKBKB
IL10RA	IL3	IKBKE
IL15	IL31RA	IL13RA1
IL17RB	IL32	IL17B
IL18	IL3RA	IL17RA
IL1A	IL4	IL6ST
IL1R1	IL5	INPPL1
IL1R2	IL5RA	IRF1
IL1RAP	IL6	IRF2
IL1RL1	IL9	IRF3
IL1RN	ITGA2B	ISG20

IL2RG	ITGA4	ITGA6
IL34	ITGB4	ITGAV
IL7	ITK	ITGB1
IL7R	KLRB1	JAK1
ILF3	KLRF1	JAK2
IRAK1	KLRK1	KMT2D
IRAK4	LCN2	KRT14
ISG15	LOC100049001	KRT18
ITGA1	LOC100683403	KRT7
ITGA2	LOC102155900	LAMP1
ITGA5	LOC102156626	LAMP2
ITGAL	LOC102156778	LCP1
ITGAM	LOC102156836	LGALS1
ITGAX	LOC111094769	LGALS3
ITGB2	LOC475935	LOC100683099
ITGB3	LOC476396	LOC102153988
JAM3	LOC476900	LOC102154078
KDM5C	LOC480600	LOC102156614
KDR	LOC484343	LOC478984
KIT	LOC490356	LOC484306
KLRG1	LOC606890	LRP1
KMT2A	LOC609023	LTF
KRT8	LOC612539	LY96
LAIR1	LTA	LYN
LAP3	LTB	MAF
LBP	LTK	MAP2K2
LFNG	MAP2	MAP3K1
LOC100686511	MAPK11	MAP3K5
LOC100856270	MASP1	MAP3K7
LOC477699	MASP2	MAPK1
LOC481722	MBL2	MAPK14
LOC487977	MEFV	MAPK3
LTBR	MR1	MAPK8
LY9	MS4A1	MAVS
MAP2K1	MS4A2	MDM4
MAP2K4	MUC1	MEF2C
MAP4K2	NCR1	MET
MAPKAPK2	NCR3	MIF
MARCO	NOD2	MKI67
MCAM	NOS2	MME
MMP1	NTRK1	MMP9

MRC1	OLIG2	MRPL19
MSR1	PAX5	MTOR
MST1R	PLA2G1B	MX1
NCAM1	PNOC	MYC
NCF4	PPBP	MYD88
NFATC1	PRF1	NDRG1
NFATC2	PTGDR2	NF1
NFATC3	PTGER2	NFKB1
NFATC4	RAG2	NFKB2
NLRC5	RORC	NFKBIA
NOD1	S100B	NOTCH1
NRDE2	SELE	NPNT
NRP1	SLAMF1	NR1H3
NUP62	SPIB	NRAS
OAS3	STAT4	NT5E
PALB2	TBC1D10B	OAS2
PDCD1LG2	TICAM1	OAZ1
PDGFRB	TIGIT	PARP1
PDPN	TLR10	PCNA
PECAM1	TLR9	PDGFC
PGR	TNFRSF13B	PDGFRA
PIK3C2B	TNFRSF4	PIK3CA
PIK3C2G	TNFRSF8	PIK3R1
PIK3CG	TNFRSF9	PIN1
PLAU	TNFSF15	PIP
PNMA1	TNFSF18	PLCG1
PPARG	TNFSF4	POLR2A
PPARGC1A	TNFSF8	PRKCD
PRKCE	TOX	PSMB9
PSMB8	TRAT1	PTEN
PSMC4	TRGC3	PTGS2
PTGER4	TRGC8	PTHLH
PTPRC	TXK	PTK2
PVR	XCR1	PUM1
RAD51	ZAP70	RAF1
RELB		RB1
RIPK2		REL
RORA		RELA
S100A12		RPS6
S100A9		RUNX1
SAA1		S100A10

SELL	S100A4
SELPLG	S100A8
SERPINB2	SDC4
SHMT2	SDHA
SIGIRR	SERPING1
SIGLEC1	SETD2
SLC11A1	SF3A1
SLC16A3	SMAD2
SMAD3	SPP1
SMARCA4	SPRY2
SOX10	ST6GAL1
SRC	STAT1
STAT5B	STAT2
SYK	STAT3
TANK	STAT5A
TBK1	STAT6
TFE3	STK11
TFEB	TAP1
TGFB1	TAPBP
THBD	TBP
THY1	TCF7
TICAM2	TFRC
TIRAP	TGFB2
TLK2	THBS1
TLR1	TLR2
TLR3	TNFRSF12A
TLR4	TNFRSF1A
TLR6	TNFRSF1B
TLR7	TNFSF10
TLR8	TNKS
TMUB2	TNKS2
TNFAIP3	TP53
TNFRSF11A	TRAF3
TNFRSF11B	TRIB1
TNFRSF13C	TSC2
TNFSF11	TXNIP
TNFSF12	TYK2
TNFSF13	TYMS
TNFSF13B	TYROBP
TOLLIP	VEGFA
TP63	VIM

TRAF1	VSIR
TRAF6	
TRBC	
TREM1	
TREM2	
TUBB3	
UBB	
VCAM1	
VEGFC	
ZEB1	
TNFRSF18	
CCND3	
ICOSLG	
CDKN2B	
CCL28	

NOTE: Please save this file locally before filling in the table, DO NOT work on the file within your internet browser as changes will not be saved. Adobe Acrobat Reader (available free here) is recommended for completion.

The ARRIVE guidelines 2.0: author checklist

The ARRIVE Essential 10

These items are the basic minimum to include in a manuscript. Without this information, readers and reviewers cannot assess the reliability of the findings.

Item		Recommendation	Section/line number, or reason for not reporting
**Study design**	1	For each experiment, provide brief details of study design including:	
		a. The groups being compared, including control groups. If no control group has been used, the rationale should be stated.	Materials & Methods section, lines 159-162
		b. The experimental unit (e.g. a single animal, litter, or cage of animals).	Materials & Methods section, lines 127-129
**Sample size**	2	a. Specify the exact number of experimental units allocated to each group, and the total number in each experiment. Also indicate the total number of animals used.	Materials & Methods section, lines 131-132, 159-162
		b. Explain how the sample size was decided. Provide details of any *a priori* sample size calculation, if done.	Not applicable
**Inclusion and exclusion criteria**	3	a. Describe any criteria used for including and excluding animals (or experimental units) during the experiment, and data points during the analysis. Specify if these criteria were established *a priori*. If no criteria were set, state this explicitly.	Materials & Methods section, lines 127-131; & Supplem. Data
		b. For each experimental group, report any animals, experimental units or data points not included in the analysis and explain why. If there were no exclusions, state so.	No exclusions
		c. For each analysis, report the exact value of *n* in each experimental group.	Materials & Methods section, lines 131-132, 159-162
**Randomisation**	4	a. State whether randomisation was used to allocate experimental units to control and treatment groups. If done, provide the method used to generate the randomisation sequence.	Materials & Methods section, lines 159-162
		b. Describe the strategy used to minimise potential confounders such as the order of treatments and measurements, or animal/cage location. If confounders were not controlled, state this explicitly.	Not applicable
**Blinding**	5	Describe who was aware of the group allocation at the different stages of the experiment (during the allocation, the conduct of the experiment, the outcome assessment, and the data analysis).	Not blinding
**Outcome measures**	6	a. Clearly define all outcome measures assessed (e.g. cell death, molecular markers, or behavioural changes).	Materials & Methods section, lines 150-154
		b. For hypothesis-testing studies, specify the primary outcome measure, i.e. the outcome measure that was used to determine the sample size.	Not applicable
**Statistical methods**	7	a. Provide details of the statistical methods used for each analysis, including software used.	Materials & Methods: lines 242-251, & Suppl. Data
		b. Describe any methods used to assess whether the data met the assumptions of the statistical approach, and what was done if the assumptions were not met.	Not applicable
**Experimental animals**	8	a. Provide species-appropriate details of the animals used, including species, strain and substrain, sex, age or developmental stage, and, if relevant, weight.	Materials & Methods: lines 127-135
		b. Provide further relevant information on the provenance of animals, health/immune status, genetic modification status, genotype, and any previous procedures.	Materials & Methods: lines 127-135, & Suppl. Data
**Experimental procedures**	9	For each experimental group, including controls, describe the procedures in enough detail to allow others to replicate them, including:	
		a. What was done, how it was done and what was used.	Materials & Methods: lines 139-241
		b. When and how often.	Materials & Methods: lines 139-241
		c. Where (including detail of any acclimatisation periods).	Materials & Methods: lines 123-127
		d. Why (provide rationale for procedures).	Discussion section: lines 467-480
**Results**	10	For each experiment conducted, including independent replications, report:	
		a. Summary/descriptive statistics for each experimental group, with a measure of variability where applicable (e.g. mean and SD, or median and range).	Tables 1, S1
		b. If applicable, the effect size with a confidence interval.	Not applicable

The Recommended Set

These items complement the Essential 10 and add important context to the study. Reporting the items in both sets represents best practice.

Item		Recommendation	Section/line number, or reason for not reporting
**Abstract**	11	Provide an accurate summary of the research objectives, animal species, strain and sex, key methods, principal findings, and study conclusions.	Abstract: lines 36-50
**Background**	12	a. Include sufficient scientific background to understand the rationale and context for the study, and explain the experimental approach.	Introduction: lines 56-108
		b. Explain how the animal species and model used address the scientific objectives and, where appropriate, the relevance to human biology.	Introduction: lines 65-73, 109-118
**Objectives**	13	Clearly describe the research question, research objectives and, where appropriate, specific hypotheses being tested.	Introduction: lines 111-114
**Ethical statement**	14	Provide the name of the ethical review committee or equivalent that has approved the use of animals in this study, and any relevant licence or protocol numbers (if applicable). If ethical approval was not sought or granted, provide a justification.	Materials & Methods: lines 125-127
**Housing and husbandry**	15	Provide details of housing and husbandry conditions, including any environmental enrichment.	Not applicable
**Animal care and monitoring**	16	a. Describe any interventions or steps taken in the experimental protocols to reduce pain, suffering and distress.	Supplem. Data
		b. Report any expected or unexpected adverse events.	Materials & Methods: lines 146-148; Results 316-317
		c. Describe the humane endpoints established for the study, the signs that were monitored and the frequency of monitoring. If the study did not have humane endpoints, state this.	Materials & Methods: lines 140-146
**Interpretation/ scientific implications**	17	a. Interpret the results, taking into account the study objectives and hypotheses, current theory and other relevant studies in the literature.	Discussion: lines 504-566
		b. Comment on the study limitations including potential sources of bias, limitations of the animal model, and imprecision associated with the results.	Discussion: lines 518-521
**Generalisability/ translation**	18	Comment on whether, and how, the findings of this study are likely to generalise to other species or experimental conditions, including any relevance to human biology (where appropriate).	Introduction: lines 65-73, 88-118; Conclusions: lines 568-574
**Protocol registration**	19	Provide a statement indicating whether a protocol (including the research question, key design features, and analysis plan) was prepared before the study, and if and where this protocol was registered.	Not applicable
**Data access**	20	Provide a statement describing if and where study data are available.	Lines 632-633
**Declaration of interests**	21	a. Declare any potential conflicts of interest, including financial and non-financial. If none exist, this should be stated.	Lines 638-643
		b. List all funding sources (including grant identifier) and the role of the funder(s) in the design, analysis and reporting of the study.	Lines 621-627
